# Adipose tissue dysfunction disrupts metabolic homeostasis: mechanisms linking fat dysregulation to disease

**DOI:** 10.3389/fendo.2025.1592683

**Published:** 2025-06-24

**Authors:** Dana Bou Matar, Mahmoud Zhra, Walid Khaled Nassar, Haifa Altemyatt, Asfiya Naureen, Nada Abotouk, Muhammad Affan Elahi, Ahmad Aljada

**Affiliations:** ^1^ Department of Physiology, College of Medicine, Alfaisal University, Riyadh, Saudi Arabia; ^2^ Department of Biochemistry and Molecular Medicine, College of Medicine, Alfaisal University, Riyadh, Saudi Arabia; ^3^ College of Medicine, Alfaisal University, Riyadh, Saudi Arabia

**Keywords:** adipose tissue dysfunction, metabolic disorders, insulin resistance, inflammatory pathways, adipokine dysregulation, oxidative stress, mitochondrial dysfunction, cellular senescence

## Abstract

**Background:**

Metabolic disease incidence continues rising globally. Adipose tissue dysfunction serves as a crucial pathophysiological mediator. We evaluate molecular mechanisms linking adipose dysfunction to metabolic dysregulation.

**Methods:**

We systematically reviewed literature on adipose biology, stress mechanisms, inflammation, and metabolic networks. Analysis prioritized methodologically robust studies from the past decade.

**Results:**

Adipose dysfunction disrupts metabolic homeostasis through complex molecular networks. Stressed adipocytes exhibit mitochondrial impairment and endoplasmic reticulum (ER) stress. These changes alter inflammatory mediators and adipokine secretion. Brown and beige adipose regulate energy balance via uncoupling protein 1 (UCP1)-mediated thermogenesis. Key transcriptional regulators, PGC-1α and PR domain containing 16 (PRDM16), control thermogenic adipocyte development. Cellular senescence contributes significantly to age-related adipose dysfunction through inflammatory secretory phenotypes. Brown fat also secretes specialized factors influencing whole-body metabolism, emphasizing adipose tissue’s endocrine function.

**Conclusion:**

Adipose dysfunction represents a critical nexus in metabolic disease pathogenesis. Cellular stress, inflammation, and metabolic dysregulation converge at this point. Novel therapies targeting thermogenic activation and cellular senescence show promise. Despite advancing mechanistic understanding, developing effective interventions remains challenging due to adipose tissue’s complex roles in systemic metabolic regulation.

## 1 Introduction

Adipose tissue (AT) dysfunction represents a central pathophysiological process in obesity-related metabolic disorders. In health, AT maintains metabolic homeostasis by dynamically responding to energy demands through regulated lipid storage and mobilization. However, factors such as chronic nutrient excess, physical inactivity, genetic predisposition, and aging can drive AT dysfunction, overwhelming its adaptive capacity (1–14).

Multiple mechanisms underlie AT dysfunction, including mitochondrial impairment, ER stress, inflammatory pathway activation, and extracellular matrix (ECM) remodeling. These changes create self-reinforcing pathological cycles that worsen tissue function and promote systemic insulin resistance (15–17). Notably, AT dysfunction manifests across diverse metabolic disorders, from obesity-associated insulin resistance to lipodystrophies, highlighting that proper adipose function, not just presence, is essential for metabolic homeostasis.

AT comprises distinct depots (white, brown, beige, and pink) with unique physiological functions and metabolic characteristics (1, 18–21). White adipose tissue (WAT) controls lipolysis through Adipose Triglyceride Lipase (ATGL) and hormone-sensitive lipase (HSL) enzymes (19), while brown adipose tissue (BAT) expresses UCP1 for thermogenesis (20). Beige adipocytes demonstrate remarkable plasticity in response to environmental signals (1).

At the molecular level, adipose dysfunction involves cellular stress responses, aberrant inflammatory signaling, and dysregulated microRNA networks. These processes collectively compromise adipocyte metabolic and endocrine functions, disrupting inter-organ communication and systemic metabolism.

This review integrates current evidence on molecular mechanisms underlying AT dysfunction in metabolic disorders, emphasizing adipose-specific signaling networks. We examine dysfunction across various conditions and analyze emerging therapeutic approaches targeting thermogenic activation and cellular senescence pathways, providing an integrated framework for understanding metabolic disease pathogenesis and identifying novel intervention strategies.

## 2 Molecular and functional organization of adipose tissue

AT plays an essential role in metabolic regulation beyond simple energy storage (22). These distinct cell types (including the adipocytes at different stages of differentiation, resident immune cell types, and vascular elements) cooperatively organize into metabolically active tissue units in the extracellular space (23). Dense vascular beds and neural inputs regulate metabolic responses at both tissue and systemic levels.

Biochemical characteristics of AT have revealed complex regulatory networks that control energy metabolism. Key enzymes such as ATGL and HSL play a role in the storage and mobilization of lipids, and their dysregulation directly contributes to metabolic disorders (22). Several elements, such as hormonal levels, diet conditions, and the interaction of certain proteins, influence these pathways. AT acts as an endocrine organ and releases many bioactive substances that affect the overall metabolism. These adipokines control appetite, energy consumption, insulin sensitivity, and inflammation responses through a variety of signal pathways. Additionally, the tissue processes several key hormones via specific enzyme systems, modifying androgens, glucocorticoids, and thyroid hormones.

Anatomical distribution significantly impacts AT function, with distinct depots exhibiting unique molecular and metabolic profiles. Recent molecular analyses have identified four categories of AT - white, brown, beige, and pink - each serving specialized physiological roles (22, 23). These depot-specific characteristics stem from the differential expression of developmental transcription factors and metabolic enzymes.

The global rise in metabolic disease prevalence has spurred research into the molecular mechanisms underlying AT dysfunction. Analysis of AT from obese subjects reveals characteristic alterations in gene expression networks controlling metabolism, inflammation, and endocrine function. Recent studies focus on depot-specific transcriptional programs and their relationship to systemic metabolic regulation.

This article explores molecular control mechanisms in AT function and metabolic disease, based on evidence from biochemical, cellular, and clinical studies. Experimental findings highlight specific transcriptional networks and signaling pathways maintaining adipose homeostasis. Analysis of these molecular mechanisms provides critical insights for developing targeted therapeutic strategies.

## 3 AT types and their distinct functions

AT functions as a complex endocrine organ with diverse roles beyond simple energy storage (24). It encompasses multiple cell types and is classified into four distinct categories: white, brown, beige, and pink AT (14). AT’s plasticity and heterogeneous nature (**Table 1**) are fundamental to its role in energy homeostasis, metabolic regulation, and disease progression (18).

**Table 1:** Integrated analysis of AT functional networks.

Adipose Type	Primary Functions	Key Mechanisms	Physiological Impact	References
WAT	• Energy Storage & Mobilization	• Stores energy as triglycerides (200,000-300,000 kcal in adults). Regulated by insulin and catecholamines. Lipolysis via HSL and ATGL.	• Primary energy reserve. Metabolic homeostasis. Thermal insulation.	(21, 25, 26)
	• Endocrine Function	• Secretes adipokines (leptin, adiponectin, resistin). Produces inflammatory mediators (TNF-α, IL-6). Processes steroid hormones.	• Appetite regulation. Insulin sensitivity. Systemic metabolism.	(21, 25, 26)
	• Structural Support	• Provides mechanical cushioning. Maintains tissue architecture. Supports vascular networks.	• Organ protection. Joint support. Body contouring.	(21, 26, 27)
BAT	• Thermogenesis	• UCP1-mediated heat production. High mitochondrial density. β-adrenergic activation.	• Temperature regulation. Energy expenditure. Metabolic efficiency.	(28–30)
	• Metabolic Regulation	• Secretes batokines (PLTP, FGF21, VEGF-A). Glucose uptake. Fatty acid oxidation.	• Systemic metabolism. Glucose homeostasis. Lipid utilization.	(28, 30, 31)
Beige Adipocytes	• Adaptive Thermogenesis	• Inducible UCP1 expression. Recruitable from WAT. Cold/β-adrenergic responsive.	• Flexible energy expenditure. Metabolic adaptation. Temperature regulation.	(29, 31, 32)
	• Metabolic Plasticity	• Browning/whitening capacity. PGC-1α activation. FGF21 responsiveness.	• Metabolic flexibility. Energy homeostasis. Stress adaptation.	(29, 31, 32)
PAT	• Lactation Support	• Mammary gland development. Milk production. Lipid synthesis/secretion.	• Offspring nutrition. Maternal metabolism. Tissue remodeling.	(21, 27, 33)
	• Hormonal Response	• Prolactin sensitivity. Oxytocin responsiveness. Pregnancy adaptation.	• Reproductive function. Metabolic adaptation. Tissue plasticity.	(21, 27, 33)

This table delineates the hierarchical organization of AT function across four distinct depot types (WAT, BAT, Beige Adipocytes, and PAT). For each tissue type, primary functions are mapped to their underlying molecular mechanisms and subsequent physiological impacts, revealing the complex interplay between local tissue activities and systemic metabolic regulation. Understanding these relationships is crucial for developing targeted therapeutic strategies for metabolic disorders, as dysfunction in any of these pathways can contribute to conditions such as obesity, diabetes, and metabolic syndrome. The parallel presentation of mechanisms across tissue types highlights both unique specializations and common regulatory themes in adipose biology. HSL, Hormone-Sensitive Lipase; ATGL, Adipose Triglyceride Lipase; TNF-α, Tumor Necrosis Factor Alpha; IL-6, Interleukin-6; BAT, Brown Adipose Tissue; UCP1, Uncoupling Protein 1; PLTP, Phospholipid Transfer Protein; FGF21, Fibroblast Growth Factor 21; VEGF-A, Vascular Endothelial Growth Factor A; Peroxisome Proliferator-Activated Receptor Gamma Coactivator 1-alpha; PAT, Pink Adipose Tissue.

### 3.1 White AT: energy storage, endocrine regulation, and metabolic homeostasis

White AT (WAT) regulates energy homeostasis through lipolysis, with ATGL and HSL mediating 95% of triglyceride catabolism (19). These enzymes exhibit complex regulation by hormonal and nutritional status (34), with disrupted lipolysis directly contributing to metabolic disorders (35, 36). WAT functions extend far beyond simple energy storage, secreting numerous bioactive adipokines including leptin, adiponectin, resistin, visfatin, Tumor necrosis factor-α (TNF-α), and various interleukins (37, 38). Each adipokine serves distinct metabolic roles. Leptin regulates appetite and energy consumption through the hypothalamic signal, while adiponectin increases insulin sensitivity and suppresses inflammation (39). Resistin and visfatin modulate glucose metabolism and immune responses (40).

WAT also processes several key hormones, transforms androgens into estrogens through aromatization activity, and mutates glucocorticoids through type 1 11β-hydroxysteroid dehydrogenase (11β-HSD1) (41). WAT metabolizes thyroid hormones, which regulate the lipogenic and lipolytic genes (42). Additionally, WAT facilitates thyroid hormone metabolism, thereby influencing lipogenic and lipolytic gene expression (42).

Structurally, WAT provides essential mechanical functions. The tissue acts as a protective cushion for internal organs, provides joint support, and generates thermal insulation. Particularly, subcutaneous WAT functions as an effective thermal buffer during cold environmental conditions.

### 3.2 Brown AT: thermogenic organ for heat production and energy expenditure

Brown AT (BAT) is characterized by the presence of UCP1 (thermogenin), a specialized mitochondrial protein that uncouples the electron transport chain from ATP synthesis by allowing protons to leak across the inner mitochondrial membrane, thereby dissipating the proton gradient as heat rather than using it for ATP production (20). Cold exposure triggers BAT activation through sympathetic nerve stimulation and β-adrenergic signaling (28). The tissue’s high metabolic activity drives systemic energy expenditure through rapid fatty acid oxidation and glucose uptake, significantly impacting basal metabolism and temperature regulation (43).

The discovery of active BAT in adult humans in 2009 fundamentally changed understanding of metabolic regulation (44). BAT’s beige/brite adipocytes display remarkable adaptation to environmental and physiological changes (45). Higher BAT activity correlates with reduced body fat mass, suggesting protective effects against obesity (46). BAT secretes specialized signaling factors - batokines - comprising peptides, metabolites, lipids, and regulatory RNAs (47). These molecules target metabolic processes in liver, heart, muscle, and WAT through multiple signaling pathways (48).

Batokines regulate whole-body metabolism by modifying glucose handling, insulin responses, and inflammatory signals (49). Key batokines include PLTP (Phospholipid Transfer Protein), FGF21 (Fibroblast Growth Factor 21), VEGF-A (Vascular Endothelial Growth Factor A), BMP8 (Bone Morphogenetic Protein 8), NRG-4 (Neuregulin 4), and IL-6, each controlling specific metabolic pathways (50–52). These factors enhance insulin sensitivity and substrate utilization across multiple tissues (53). Type 2 diabetes mellitus (T2DM) progression alters both batokine production and BAT function (54). Recent work identifies exosomal microRNAs as additional metabolic regulators (49). IL-6 shows tissue-specific effects - promoting glucose homeostasis and energy expenditure in BAT while potentially causing insulin resistance elsewhere (55, 56). Through these diverse endocrine functions, BAT emerges as a central coordinator of systemic metabolism.

### 3.3 Beige adipocytes: inducible thermogenic cells for metabolic flexibility

Beige adipocytes are a unique thermogenic cell population that emerge within WAT through a process called “browning” or “beiging,” triggered by various environmental stimuli such as cold exposure, β-adrenergic activation, and certain hormonal signals (1). These cells exhibit significant metabolic plasticity, expressing UCP1 and transitioning between energy storage and expenditure phenotypes in response to physiological demands (57). Beige adipocytes contribute to adaptive thermogenesis through both UCP1-dependent mechanisms and alternative pathways, such as calcium cycling and creatine-driven substrate cycling, thereby enhancing glucose homeostasis and lipid metabolism (58).

The molecular pathways governing beige adipose cells utilize specific transcriptional controllers for development and function. Each factor serves distinct roles: PPAR gamma coactivator 1 alpha (PGC-1α) initiates metabolic programs, PRDM16 (PR domain containing 16) modifies chromatin structure to enable differentiation, and FGF21 (fibroblast growth factor 21) enables heat production in mature cells (59). Recent studies demonstrate how muscle-derived myokines produced during exercise influence beige fat development. Thyroid hormone signaling pathways provide additional mechanisms for regulating beige adipocyte formation and activity (60). Systematic screening has identified numerous circulating molecules that control differentiation and thermogenic capacity in both brown and beige adipocytes, including irisin, FGF21, BMP8, NRG-4, and IL-6, establishing potential therapeutic targets for treating metabolic disorder (61).

### 3.4 Pink AT: dynamic plasticity in mammary gland development and lactation

Pink AT (PAT) showcases remarkable plasticity, forming from subcutaneous WAT during pregnancy and lactation. PAT is uniquely composed of mammary gland alveolar epithelial cells, known as pink adipocytes, which are specialized for milk production and secretion (21). PAT formation occurs through transdifferentiation, where white adipocytes undergo significant phenotypic and functional changes, including the development of milk-producing capabilities and alterations in lipid storage and secretion (21). Pink adipocytes possess distinct features that differentiate them from both white and brown adipocytes, including cellular machinery and molecular pathways specialized for lactation. The transdifferentiation process involves extensive genetic reprogramming, resulting in significant shifts in cellular identity and function (62, 63). Research into PAT and its transformation mechanisms provides valuable insights into adipose biology and cellular plasticity. Understanding these processes could lead to new therapies for metabolic disorders and breast cancer by leveraging cellular reprogramming and tissue adaptability.

## 4 Molecular mechanisms of AT dysfunction

### 4.1 Cellular stress pathways

Adipocyte dysfunction manifests through multiple disrupted cellular pathways (**Table 2**). Precise cellular changes compromise lipid metabolism, glucose transport, and inflammatory responses, generating cascading effects that extend beyond the local tissue environment (1–14). Recent research into obesity-associated adipose pathology has identified several key molecular signatures. Adipocyte hypertrophy, with cell diameters expanding up to 150-200 μm (94), leads to reduced oxygen availability and tissue hypoxia (95). This is accompanied by persistent inflammatory activation, marked by elevated levels of specific cytokines, including TNF-α, IL-6, and interleukin-1β (IL-1β) (96). Laboratory analyses reveal that these cellular perturbations alter essential signaling networks, as evidenced by abnormal adipokine profiles and increased immune cell presence. The subsequent release of free fatty acids (FFAs) into circulation impairs insulin signaling across multiple peripheral tissues (4–9).

**Table 2:** Integrated analysis of adipocyte dysfunction: from molecular mechanisms to systemic impact.

Pathway	Primary Alterations	Molecular Changes	Systemic Consequences	References
Lipid Metabolism	• Enhanced lipolysis • Impaired lipogenesis • Reduced fatty acid oxidation	• Increased HSL and ATGL activity • Decreased lipogenic enzyme expression • Mitochondrial dysfunction	• Elevated circulating FFAs • Ectopic fat deposition • Systemic lipotoxicity	(19, 35, 36)
Glucose Homeostasis	• Reduced glucose uptake • Impaired insulin signaling • Decreased GLUT4 translocation	• Reduced IR activation • Impaired IRS-1/2 phosphorylation • Defective PI3K/AKT signaling	• Hyperglycemia • Insulin resistance • Metabolic inflexibility	(64–66)
Inflammatory Status	• Enhanced pro-inflammatory signaling • Immune cell infiltration • Altered adipokine secretion	• Increased NF-κB activation • Elevated TNF-α, IL-6, IL-1β • Macrophage polarization to M1 state	• Chronic inflammation • Systemic insulin resistance • Tissue dysfunction	(67–70)
Oxidative Stress	• Increased ROS production • Impaired antioxidant defenses • Mitochondrial dysfunction	• Enhanced NOX4 activity • Reduced SOD and catalase • Compromised electron transport chain	• Cellular damage • Accelerated aging • Metabolic dysfunction	(71–76)
Protein Homeostasis	• ER stress activation • Impaired protein folding • UPR activation	• BiP/GRP78 upregulation • PERK/IRE1α/ATF6 activation • Enhanced CHOP expression	• Cell death • Inflammation • Metabolic disruption	(77–80)
Extracellular Matrix	• Enhanced fibrosis • Altered matrix composition • Modified tissue mechanics	• Increased collagen deposition • Enhanced MMP activity • Modified integrin signaling	• Reduced tissue plasticity • Impaired adipogenesis • Mechanical stress	(81–83)
Cell Death Pathways	• Enhanced apoptosis • Increased pyroptosis • Necrotic cell death	• Caspase activation • Inflammasome activation • Loss of membrane integrity	• Tissue dysfunction • Chronic inflammation • Metabolic deterioration	(84–86)
Mitochondrial Function	• Reduced ATP production • Impaired fatty acid oxidation • Disrupted fusion/fission	• Decreased respiratory capacity • Modified mitochondrial dynamics • Altered metabolic flexibility	• Energy deficit • Impaired thermogenesis • Metabolic dysfunction	(87–90)
Cell Signaling	• Modified hormone responses • Altered growth factor signaling • Disrupted metabolic regulation	• Impaired insulin/leptin signaling • Modified AMPK activation • Altered mTOR signaling	• Hormone resistance • Growth dysregulation • Metabolic imbalance	(91–93)

This table systematically maps the major pathways disrupted in adipocyte dysfunction, tracking changes from molecular mechanisms to systemic consequences. The key findings are coordinated disturbances of lipid and glucose metabolism, with increased lipolysis and insulin signaling as the main drivers of metabolic deterioration. The analysis reveals complex relationships between oxidative stress, mitochondrial dysfunction and inflammation activation, creating self-insufficiency cycles of cellular stress. Notably, these pathways demonstrate significant cross-talk, where dysfunction in one system often amplifies perturbations in others. Standard field abbreviations are used throughout: FFAs, GLUT4 (glucose transporter 4), TNF-α, IL-6/1β, ROS (reactive oxygen species), UPR (unfolded protein response), and ECM (extracellular matrix). Understanding these interconnected pathways has direct therapeutic implications, suggesting potential intervention points for treating metabolic disorders. HSL, Hormone-Sensitive Lipase; ATGL, Adipose Triglyceride Lipase; IR, Insulin Receptor; IRS-1/2, Insulin Receptor Substrate-1/2; GLUT4, Glucose Transporter Type 4; PI3K, Phosphoinositide 3-Kinase; AKT, Protein Kinase B; NF-κB, Nuclear Factor Kappa-light-chain-enhancer of activated B cells; TNF-α, Tumor Necrosis Factor Alpha; IL-6, Interleukin-6; IL-1β, Interleukin-1 Beta; NOX4, NADPH Oxidase 4; SOD, Superoxide Dismutase; BiP, Binding Immunoglobulin Protein; GRP78, Glucose-Regulated Protein 78; PERK, Protein kinase R-like Endoplasmic Reticulum Kinase; IRE1α, Inositol-Requiring Enzyme 1 Alpha; ATF6, Activating Transcription Factor 6; CHOP, C/EBP Homologous Protein; AMPK, AMP-activated Protein Kinase; mTOR, Mechanistic Target of Rapamycin.

Affected adipose depots develop sustained mitochondrial defects and heightened oxidative stress, which amplify existing metabolic disruptions (10). The location of adipose expansion critically influences disease progression, with visceral and ectopic fat deposits having particularly detrimental effects on systemic metabolism (11).

### 4.2 ER stress and the unfolded protein response

The ER contains numerous quality control pathways that regulate protein processing and maintain cellular homeostasis through distinct molecular mechanisms. Binding immunoglobulin protein (BiP) requires ATP for its chaperone function, recognizing exposed hydrophobic regions on unfolded proteins to prevent aggregation. X-box binding protein 1 (XBP1) transcriptionally regulates protein folding genes, while the unfolded protein response (UPR) activates when protein folding demands increase, reducing protein synthesis while expanding folding capacity (77).

The major UPR transducers operate through biochemically distinct mechanisms. Protein kinase R-like endoplasmic reticulum kinase (PERK) activation triggers eukaryotic initiation factor 2 alpha (eIF2α) phosphorylation specifically at serine 51, leading to selective mRNA translation despite global protein synthesis attenuation. The membrane-bound transcription factor ATF6 requires sequential proteolysis within Golgi compartments, generating an active nuclear form that transcriptionally upregulates ER-resident chaperones. The bifunctional enzyme inositol-requiring enzyme 1 α (IRE1α) exhibits both protein kinase and site-specific endoribonuclease activities, enabling XBP1 mRNA processing and targeted decay of ER-associated transcripts via Regulated IRE1-Dependent Decay (RIDD) (78). Analysis of metabolically compromised tissues reveals pronounced activation of these UPR components, particularly within AT, where UPR activation is significantly increased, with insulin upregulating the UPR dose-dependently over the entire physiological insulin range (from approximately 35 to 1,450 pmol/L) (97).

In AT, obesity induces pronounced ER stress that fundamentally disrupts metabolic homeostasis through multiple tissue-specific mechanisms (80, 98). Proteomic analyses of obese AT reveal significant upregulation of UPR-related proteins including calreticulin and protein disulfide-isomerase A3, with Glucose-Regulated Protein 78 (GRP78)/BiP expression increased 3–4 fold compared to lean controls (99, 100). This AT-specific ER stress directly impairs insulin signaling by promoting c-Jun N-terminal kinase (JNK)-mediated serine phosphorylation of insulin receptor substrate (IRS-1), reducing glucose uptake capacity by up to 60% (101). Moreover, UPR activation in adipocytes dramatically alters adipokine production patterns, with adiponectin secretion decreased while inflammatory cytokines including IL-6 and resistin are increased (101, 102).

The NOD-like receptor family, pyrin domain containing 3 (NLRP3) inflammasome represents a critical mediator of AT dysfunction during ER stress. Activated by lipotoxicity and ER stress signals, NLRP3 triggers IL-1β and IL-18 release specifically in adipocytes, promoting tissue inflammation and macrophage recruitment (103, 104). TNFα-induced NLRP3 activation in adipocytes causes mitochondrial dysfunction and exacerbates insulin resistance, while also impairing white adipocyte browning and thermogenic capacity (105, 106). The Toll-like receptor 4 (TLR4)/PI3K/Akt pathway converges with ER stress responses to amplify AT inflammation (107). Importantly, caloric restriction and exercise reduce AT NLRP3 expression and inflammation, suggesting therapeutic potential (104).

AT-specific UPR activation disrupts lipid metabolism through PERK-ATF4 pathway signaling. ATF4 regulates thermogenesis and lipolysis by influencing fatty acid utilization gene expression (108). The three UPR sensors - IRE1, PERK, and ATF6 - alter lipid enzyme function in adipocytes, affecting fatty acid synthesis and oxidation (109, 110). High-fat diets intensify ER stress in AT, compromising endocrine function and accelerating metabolic disease progression (78). The ER stress-induced transcription factor C/EBP homologous protein (CHOP) further drives AT dysfunction by promoting M1 macrophage polarization while suppressing anti-inflammatory Th2 cytokines (111).

Prolonged UPR activation fundamentally alters cellular function despite its initial adaptive purpose. Extended perturbation of ER function progressively compromises insulin biosynthetic capacity and ultimately triggers CHOP-dependent apoptotic programs through calcium-dependent mechanisms and mitochondrial dysfunction (112). These molecular alterations characterize both obesity and diabetes, where chronic ER dysfunction perpetuates inflammatory signaling networks and impairs IR signal transduction through multiple intersecting pathways (79, 80).

IRE1α activates JNK through TNF receptor-associated factor (TRAF2) binding, triggering inflammatory signaling cascades. Nuclear factor kappa-light-chain-enhancer of activated B cells (NF-κB) activation occurs through both PERK and IRE1α pathways, increasing inflammatory cytokine production. These pathways create a feed-forward cycle where altered lipid metabolism amplifies cellular stress and inflammation (79, 80). The ER integrates nutrient sensing across cell types through regulation of calcium fluxes and lipid metabolic programs, coordinating cellular responses to metabolic fluctuations (113). Post-transcriptional control through microRNA networks, including miR-211, miR-30c, and miR-34a, modulates the cellular response to protein folding stress, fine-tuning both adaptive and maladaptive responses (114). Experimental manipulation of ER protein folding capacity influences beige adipocyte differentiation programs and metabolic adaptation to high-fat feeding through coordinated effects on mitochondrial homeostasis and inflammatory tone in multiple tissues (115).

Therapeutic targeting of AT ER stress shows promise through chemical chaperone interventions. Studies utilizing 4-phenylbutyrate demonstrate reductions in adipose GRP78 expression concurrent with decreased plasma metabolites. Biochemical evaluation reveals 4-PBA mediated suppression of PERK and IRE1α phosphorylation cascades while maintaining IRS-1 function (116). Investigation of small molecule UPR modulators has yielded synergistic effects, with the selective IRE1α inhibitor HT-6184 enhancing semaglutide-mediated improvements in body composition and glucose homeostasis. Combined administration produces superior outcomes with enhanced preservation of lean mass and decreased ceramide accumulation in adipose depots (117). These findings establish AT ER stress as a central mechanism linking obesity to systemic metabolic disorders including T2DM and cardiovascular disease (118, 119).

### 4.3 The role of mitochondria in AT dysfunction and metabolic disorders

AT biopsies reveal substantial mitochondrial alterations at both structural and functional levels. Quantitative analysis of oxygen consumption demonstrates a 45% reduction in oxidative phosphorylation capacity, accompanied by dysregulated acyl-CoA oxidation and accumulation of oxidative stress markers, specifically 4-hydroxynonenal adducts within subcutaneous adipose deposits (87). Mass spectrometry-based metabolomic analyses have identified distinct fatty acid profiles characteristic of pathological tissue states.

ChIP-seq analysis has mapped PGC-1α binding sites across nuclear respiratory gene promoters, establishing direct transcriptional control mechanisms. Clinical specimens exhibit marked metabolic protein dysregulation - PGC-1α expression decreases 65% compared to controls, concurrent with diminished UCP1 and Carnitine palmitoyltransferase 1 (CPT1) protein levels as determined by immunoblot analysis (88). Tissue-specific deletion of PGC-1α in adipose reveals its metabolic regulatory functions. Adipose-selective PGC-1α ablation induces rapid insulin resistance alongside decreased thermogenic gene expression and mitochondrial dysfunction (120). This nuclear coactivator responds to metabolic signals to regulate oxidative phosphorylation genes through interactions with key transcription factors (121). Studies in WAT demonstrate PGC-1α’s requirement for both baseline and rosiglitazone-stimulated mitochondrial activity, though insulin sensitization by thiazolidinediones (TZDs) persists in its absence (122). Global PGC-1α knockout mice display unexpected metabolic phenotypes - reduced adiposity and increased physical activity despite compromised mitochondrial capacity (123). Moderate elevation of PGC-1α within physiological ranges augments fatty acid oxidative capacity and glucose transport in response to insulin (124). In cardiac tissue, PGC-1α collaborates with PGC-1β to preserve mitochondrial function as insulin resistance develops (125). Metabolic phenotyping studies demonstrate depot-specific requirements for PGC-1α, particularly in BAT where its absence severely impairs thermogenic capacity. PGC-1 coactivators regulate metabolism through coordinated binding to nuclear receptors, enabling adaptive responses to nutritional status and environmental cues. These molecular interactions establish distinct transcriptional programs across tissues to maintain metabolic homeostasis.

Dynamic imaging reveals fragmented mitochondrial networks and accelerated ROS generation in metabolically compromised adipocytes. Longitudinal clinical studies demonstrate progressive decline in PGC-1α activity correlating with heightened inflammatory markers and oxidative damage in AT specimens (120, 126). These mechanistic findings from both human pathology and experimental models highlight the therapeutic relevance of mitochondrial quality control mechanisms.

Biochemical analyses establish AMPK as a central regulator of mitochondrial biogenesis through direct modulation of lipid oxidation. Phosphoproteomic mapping in cardiac tissue reveals extensive AMPK-dependent signaling networks, where pathway perturbation produces severe metabolic consequences (16). Current therapeutic development focuses on mitochondrial function, with particular success seen in thiazolidinedione compounds that upregulate PGC-1α and mitochondria-targeted antioxidants (89, 90). Excessive mitochondrial ROS, especially superoxides (O2^•-^), hydrogen peroxides (H_2_O_2_) and hydroxides (^•^OH), characterize obesity adipocyte dysfunction. These cells show a significant reduction in antioxidant defense capacity, and reactive oxygen species (ROS) concentrations in cells usually go from physiological (100nM H_2_O_2_) to pathological (>500nM H_2_O_2_) (97, 98). NADPH oxidase 4 (NOX4) transfer electrons to molecular oxygen and acts as the main ROS generator. Redox homeostasis is controlled by a variety of cell defense mechanisms, including three different superoxide dismutase (SOD1 in cells, SOD2 in mitochondria, SOD3), glutathione peroxidase (GPX), and signal pathways Nuclear factor erythroid 2-related factor 2 (NRF2)- Kelch-like ECH-associated protein 1(KEAP1) (71).

Oxidative damage disrupts the potential of the mitochondrial membrane and inhibits the function of the I-IV electron transport chain. Due to the lack of control of fusion proteins (Mitofusin-1/2(MFN1/2), Optic atrophy 1(OPA1)) and fission proteins (Dynamin-related protein 1 (DRP1), mitochondrial fission 1 protein (FIS1), ROS accumulation changes the dynamics of mitochondrial networks (72–75). These changes reduce respiratory capacity by 50% and inhibit adipogenic differentiation (127, 128). The resulting metabolic disturbances trigger the production of adipocin, especially TNF-α, IL-6, and Monocyte Chemoattractant Protein-1 (MCP-1, also known as CCL2), to induce inflammatory mediators.

Cellular ROS concentrations require precise regulation through a complex interplay with calcium signaling and ER function. Both excessive and insufficient oxidative states compromise adipocyte function (76). Mitochondrial ROS serve as critical signaling molecules, regulating mitochondrial DNA transcription and modulating UCP1-dependent thermogenesis. These pathways directly influence cellular bioenergetics, with multiple metabolic processes depending on proper ROS signaling. Energy expenditure patterns show ROS-dependent regulation through AMPK pathway activation (129).

AT in obesity displays persistent inflammatory activation, with oxidative stress mechanisms operating continuously. These processes establish feed-forward cycles of metabolic dysfunction (130, 131). Multiple cellular ROS sources contribute to oxidative burden, with mitochondrial electron transport generating approximately 70% of total cellular ROS. NADPH oxidase complexes produce the remaining oxidative species, which activate NF-κB-dependent transcriptional programs (132). This activation results in a 5–10 fold increase in pro-inflammatory cytokine production.

Metabolic perturbations impair IR signaling cascades through JNK and IκB kinase-β (IKKβ) activation, leading to serine phosphorylation of IRS-1. They dysregulate lipid metabolism and storage, contributing to cardiovascular pathologies. Adipocyte hypertrophy triggers immune cell recruitment, with M1 macrophage infiltration maintaining the inflammatory state (133). Multiple pathological triggers initiate these cascades, including dietary lipid excess and altered microbiota composition, particularly reduced Bacteroidetes-to-Firmicutes ratio.

Oxidative stress synergizes with inflammatory processes in AT to severely disrupt glucose homeostasis. ROS directly modify insulin signaling proteins through oxidation of critical cysteine residues. Oxidative damage reduces glucose transporter type 4 (GLUT4) vesicle translocation by approximately 60%, as measured by membrane fraction analysis (133). Cellular glucose uptake capacity diminishes markedly, typically showing a 70-80% reduction compared to healthy adipocytes.

Current interventions target multiple pathways simultaneously for maximal effect. Currently, certain NOX4 inhibitors (GKT137831), as well as mitochondrial antioxidants (MitoQ, SS-31), of which some are in phase II clinical trials (134), fall into this category. Lifestyle changes, especially adherence to regular physical exercise and Mediterranean diet-as reflected by reduced oxidative stress of adipocytes (as measured by plasma levels of F2-isoprostanes and carbonyls of proteins)-showed positive effects (135, 136). Some of the important biomarkers to monitor are systemic oxidative stress (8-isoprostane, malondialdehyde), antioxidant capacity (Reduced glutathione/Oxidized glutathione (GSH/GSSG) ratios, SOD activity), inflammation (high-sensitivity C-reactive protein (CRP), IL-6), and metabolic function (adiponectin/leptin ratios) (137, 138).

Novel therapeutic approaches involve mitochondrial-targeted antioxidants, combination anti-inflammatory and antioxidant strategies, ER stress modulators, and microbiote-targeted therapies. These multi-targeted interventions try to restore metabolic homeostasis through multiple mechanisms targeting oxidative stress, inflammation, and cell metabolism simultaneously.

### 4.4 Inflammatory signaling

#### 4.4.1 Immune cell infiltration in AT: mechanisms, consequences, and clinical implications

AT in obesity shows chronic low-grade inflammation. Multiple factors drive this inflammatory state. Enlarged adipocytes interact with infiltrating immune cells. Cellular stressors like mechanical strain from hypertrophy and local hypoxia initiate AT inflammation. Under stress, adipocytes release pro-inflammatory signals. MCP-1 acts as a key mediator that recruits immune cells to the tissue (139, 140). Macrophages are the first to respond, triggering self-perpetuating inflammatory cycles that increase cytokine production and alter tissue structure (133). These inflammatory changes progressively disrupt normal metabolism, leading to insulin resistance and numerous obesity complications (141). Adipocytes and immune cells establish complex interactions through adipokines and cytokines, affecting lipolysis regulation and maintaining a state of chronic inflammation in the tissue (142).

AT shows defined patterns as disease progresses (143). M2 macrophages shift to M1 type, gathering near dying fat cells. These M1 cells make crown structures and release TNF-α, IL-1β, and IL-6 (144, 145). Over time, these changes impair metabolism and cause insulin resistance (107). Multiple interconnected factors drive this dysfunction: continuing adipocyte hypertrophy, worsening tissue hypoxia, and elevated circulating FFAs create self-reinforcing inflammatory cycles. These changes result in increasingly severe metabolic dysregulation throughout the tissue (146).

The immune cell population in dysfunctional AT extends far beyond macrophages. The tissue accumulates CD8+ T cells, Th1 cells, natural killer cells, B cells, neutrophils, and mast cells in significant numbers (147). This diverse immune network amplifies inflammatory signaling through multiple pathways that disrupt normal AT function, impair insulin signaling cascades, and dramatically alter adipokine production patterns (133, 148). Ongoing inflammation changes tissue structure through fibrosis and altered function (149). Levels of MCP-1, C-C motif chemokine ligand 5/Regulated upon Activation, Normal T Cell Expressed and Secreted (CCL5/RANTES), IL-6, IFN-γ (interferon-γ), and TNF-α increase in the tissue (145). The effects reach beyond AT and disrupt multiple organ systems (133). Both B and T lymphocytes play essential regulatory roles in tissue inflammation and insulin sensitivity (150). The coordinated activities of macrophages, T cells, and NK cells drive substantial tissue remodeling and promote widespread metabolic dysfunction (151, 152).

#### 4.4.2 Pro-inflammatory mediators

Pro-inflammatory Mediators Obesity-induced inflammation in AT involves macrophage infiltration and stress pathway activation, with NF-κB driving production of cytokines (TNF-α, IL-6, IL-1β) that disrupt insulin signaling (67, 68). Notably, C-C chemokine receptor type 2 (CCR2)-expressing M1 macrophages preferentially accumulate around necrotic adipocytes rather than arising through phenotypic conversion of resident M2 populations (68). Advanced single-cell RNA sequencing has uncovered previously unidentified AT macrophage subpopulations that transcend the traditional M1/M2 dichotomy (153). Mass spectrometry-based proteomic profiling has identified specific adipokine signatures that regulate macrophage chemotaxis and inflammatory activation states (140).

The progression of obesity involves multiple factors driving macrophage phenotypic alterations. Elevated FFAs activate inflammatory signaling cascades while complex cytokine networks orchestrate cellular responses (154, 155). The accumulation of M1 macrophages triggers activation of JNK and IKK pathways through pattern-recognition receptor engagement, promoting insulin resistance through stress kinase signaling mechanisms (69, 70). This process is exacerbated by concurrent reductions in anti-inflammatory mediators, particularly adiponectin, further disrupting AT metabolic homeostasis (156). Early immunological alterations include depletion of regulatory immune cell populations alongside enhanced activation of natural killer cells and CD8+ T lymphocytes (157). These inflammatory mechanisms progressively compromise multiple organ systems, accelerating the development of metabolic disease (5).

#### 4.4.3 Chronic inflammation in AT: pathways to metabolic dysfunction

Self-perpetuating inflammatory circuits establish chronic AT dysfunction through interconnected molecular pathways. Initial inflammatory responses triggered by adipose expansion become self-sustaining through multiple feedback loops. The effective therapeutic targeting of these inflammation networks requires a detailed understanding of the pathological contributions to metabolic deterioration. Current research continues to uncover new regulatory mechanisms with potential therapeutic applications for obesity-related metabolic disorders. The development of targeted anti-inflammatory interventions may provide promising strategies to restore AT homeostasis.

### 4.5 Metabolic dysregulation

#### 4.5.1 Insulin signaling pathways in obesity and metabolic disease

Metabolic tissues develop lipid intermediates alongside altered adipokine expression profiles and compromised mitochondrial function, progressively attenuating insulin responsiveness (64, 65). These changes compromise glucose homeostasis and create more widespread metabolic derangements associated with insulin-resistant conditions (158). Adipocytes use mechanistic target of rapamycin complex 1 (mTORC1) and mTORC2 for metabolic control (91). These complexes regulate the activities of IRS-1 and the growth factor receptor-bound protein 10 (Grb10) and fine-tune their insulin responses, from complex feedback circuits that ensure metabolic homeostasis in different nutrition environments (92). In AT, mTOR mediates multiple cellular processes via various downstream effectors controlling cellular growth, differentiation, and metabolic homeostasis. Conditional deletion of mTOR specifically from AT leads to decreased fat mass with increased systemic insulin resistance and hepatic lipid deposition (159).

In adipocytes, mTORC2 regulates both insulin-stimulated glucose transport and lipolysis rates (160). This regulation is achieved through Akt substrate of 160kDa (AS160) that produces cellular responses to both Akt and mTORC2 signals to regulate glucose transporter 4 (GLUT4) trafficking during insulin stimulation, and to maintain glucose homeostasis (161). The mTOR dependent mechanisms described give insights into selective insulin resistance in adipocytes and propose novel therapeutic strategies for interventions in the metabolic disease.

Inflammatory signaling via TNF-α disrupts insulin response pathways at multiple levels. TNF-α modifies IRS-1 through enhanced serine phosphorylation, inhibiting the tyrosine phosphorylation events essential for normal insulin signal transduction (162, 163). IκB kinase (IKK) phosphorylates specific serine residues on IRS-1, maintaining the insulin-resistant state throughout the inflammatory response (164). Suppressor of cytokine signaling (SOCS) proteins, most notably the SOCS-3, inhibit insulin signaling by preventing IRS protein activation through direct molecular interactions and altered protein stability in metabolically active tissues (165). TNF-α and SOCS3 establish a positive feedback loop that amplifies their expression and intensifies insulin resistance through sustained inflammatory pathway activation in AT (166) (**Table 3**). These molecular connections between inflammation and insulin resistance identify specific therapeutic targets for addressing metabolic disorders and their complications in obesity (93).

**Table 3:** Molecular mechanisms of insulin signaling disruption in AT.

Mechanism/Protein	Expression/Activity	Consequences	Metabolic impact on AT	Regulatory influence	References
IR	Reduced function with inflammatory serine phosphorylation	Suppressed tyrosine phosphorylation; impaired signal transduction	Decreased glucose uptake; disrupted insulin signal initiation	Inflammatory cytokines (TNF-α) directly block receptor activation	(162–165)
IRS-1	Increased inhibitory serine phosphorylation	Impaired tyrosine phosphorylation	Disrupted insulin signaling cascade	Directly inhibited by IKK complex and inflammatory mediators	(66, 162–164)
PI3K/Akt Pathway	Diminished activation	Reduced insulin-stimulated glucose disposal	Metabolic inflexibility; decreased energy metabolism	Upstream signaling impairment leads to downstream effector modulation	(66, 91, 161)
GLUT4	Reduced expression and membrane translocation	Impaired glucose transport	Significantly decreased glucose uptake efficiency	Regulated by insulin signaling and inflammatory processes	(167, 168)
SNARE Proteins (VAMP2, Syntaxin 4, SNAP23)	Disrupted complex formation and interaction	Compromised vesicle fusion	Impaired glucose transporter trafficking	Directly influenced by insulin signaling and inflammatory mediators	(169–171)
Rab GTPases	Altered targeting specificity and activation	Inefficient GLUT4 trafficking	Reduced glucose uptake precision	Modulated by AS160/Tbc1D4 and upstream signaling pathways	(172–174)
AS160/Tbc1D4	Impaired phosphorylation and regulatory function	Disrupted Rab GTPase modulation	Compromised GLUT4 translocation	Critically regulated by Akt and insulin signaling cascade	(66, 161, 174)
mTORC1/mTORC2	Dysregulated complex activity	Disrupted metabolic homeostasis	Altered cellular development and metabolism	Central regulatory hub for insulin sensitivity	(91, 159, 160)
TNF-α	Elevated inflammatory signaling	Induces serine phosphorylation of key signaling proteins	Systemic inflammatory interference	Creates positive feedback loop with SOCS3	(162, 163, 166)
IL-6	Elevated levels	Inhibits IR signaling; induces serine phosphorylation of IRS-1	Impaired glucose metabolism; increased hepatic glucose output	Produced by AT and muscle; contributes to systemic inflammation	(55, 56, 175)
SOCS3	Increased expression	Inhibits IRS protein tyrosine phosphorylation	Amplifies insulin resistance	Suppresses insulin signaling through multiple mechanisms	(165, 166)

An overview of key proteins and pathways mediating insulin resistance in AT. Listed mechanisms include IR serine phosphorylation states, IRS-1 modifications, PI3K/AKT pathway attenuation, GLUT4 expression changes, SNARE complex assembly defects, Rab GTPase cycling alterations, AS160/Tbc1D4 dysregulation, mTORC1/mTORC2 signaling perturbations, and inflammatory mediator effects (TNF-α, SOCS3, IL-6). Each component’s contribution to glucose transport dysfunction and metabolic disruption is specified. Downstream consequences on vesicle trafficking, membrane fusion, and glucose disposal are detailed. The table highlights both direct insulin signaling impairments and inflammatory pathway interactions that amplify insulin resistance. IR, Insulin Receptor; IRS-1, Insulin Receptor Substrate-1; PI3K/Akt Pathway, Phosphoinositide 3-Kinase/Protein Kinase B; GLUT4, Glucose Transporter Type 4; SNARE, Soluble NSF Attachment Protein Receptor; VAMP2, Vesicle-Associated Membrane Protein 2; SNAP23, Syntaxin 4, Synaptosomal-Associated Protein 23; Rab GTPases, AS160/Tbc1D4, Akt Substrate of 160kDa/TBC1 Domain Family Member 4; mTORC1/mTORC2, Mechanistic Target of Rapamycin Complex 1/2; TNF-α, Tumor Necrosis Factor Alpha; IL-6, Interleukin-6; SOCS3, Suppressor of Cytokine Signaling 3.

#### 4.5.2 Impaired glucose metabolism in AT

The adipose inflammatory milieu generates self-perpetuating cycles of metabolic deterioration that progressively advance insulin resistance and T2DM pathogenesis. Obesity-induced inflammation in AT leads to insulin resistance through various mechanisms. Proinflammatory macrophages secrete cytokines and microvesicles that impair insulin signaling and glucose uptake in adipocytes (176–178). This results in decreased GLUT4 translocation and reduced insulin-stimulated glucose uptake (167). Neuregulin 4 downregulation in adipocytes induces insulin resistance through inflammation and autophagic degradation of GLUT4 vesicles (179). The inflammatory milieu in obese AT disrupts normal function and leads to systemic insulin resistance (10).

Insulin-stimulated glucose uptake in AT involves the translocation of GLUT4 transporters from intracellular vesicles to the plasma membrane, mediated by soluble NSF attachment protein receptor (SNARE) proteins (168). The t-SNAREs syntaxin4 and synaptosomal-associated protein 23 (SNAP23) are essential for tethering GLUT4 vesicles to the plasma membrane, while the v-SNARE vesicle-associated membrane protein 2 (VAMP2) is crucial for fusion (169). These SNARE proteins are localized in lipid rafts, which may serve as platforms for GLUT4 vesicle fusion (170). Insulin stimulation increases syntaxin4-containing SNARE complex formation, possibly through phosphorylation of the regulatory protein Munc18c (171). Other regulatory factors, such as Rab GTPases, contribute to targeting specificity in the GLUT4 secretory pathway (172).

The insulin signaling network in metabolically compromised adipocytes manifests multiple molecular defects: impaired IR activation kinetics, substantial reduction in IRS-1 tyrosine phosphorylation, marked suppression of PI3K/Akt pathway signal propagation, and pathological elevation of inhibitory serine phosphorylation events on IRS-1 (66). These coordinated molecular perturbations manifest physiologically as systemic glucose disposal deficits, compensatory hyperinsulinemia, and progressive advancement toward metabolic syndrome.

Comprehensive understanding of these molecular regulatory networks offers critical insights for therapeutic development in insulin resistance and T2DM (**Table 3**).

### 4.6 Metabolic disruptions in lipid homeostasis

Peroxisome proliferator-activated receptor gamma (PPARγ) determines adipocyte identity while coordinating with sterol regulatory element-binding protein (SREBP1c) to regulate lipid homeostasis (180). ATGL and HSL mediate triglyceride breakdown through sequential enzymatic actions (181). Entry of fatty acids into cells requires cluster of differentiation 36 protein, followed by fatty acid binding protein 4-mediated transfer between cellular compartments. Metabolic disease states trigger enhanced activity of both lipases in AT, thereby increasing systemic fatty acid levels (182). Complex mechanisms control these changes through transcriptional regulation and post-translational modifications that alter protein function, while protein-protein interactions coordinate responses (183).

Insulin suppresses lipolysis through multiple mechanisms, with acute signaling cascades responding rapidly and transcriptional regulation occurring more slowly. ATGL expression shows particular insulin sensitivity (184). Perilipin proteins coat lipid droplets and regulate lipid storage tightly, with fatty acid release depending on perilipin function (185). Disrupted lipolytic balance promotes disease, as obesity develops progressively, leading to insulin resistance and frequently resulting in T2DM (186). Concurrent with enhanced lipolysis, adipocyte dysfunction involves impaired lipogenic capacity. PPARγ functions as a master regulator of adipocyte function and lipid homeostasis (187), with PPARγ2 specifically controlling AT lipid storage and metabolic flexibility (188). sterol regulatory element-binding protein 1c (SREBP1c) works in concert with PPARγ to regulate lipogenic gene expression, while fatty acid binding protein 4 (FABP4) and Cluster of differentiation 36 (CD36) facilitate fatty acid transport and metabolism. Compromised PPARγ function results in decreased expression of lipolytic genes and abnormal catecholamine-induced lipolysis (189). As AT serves as a critical buffer for daily lipid flux, its dysfunction can lead to ectopic fat accumulation and insulin resistance (190). PPARγ activation enhances AT function by modifying fat distribution, adipocyte phenotype, and lipid metabolism-related gene expression (191). Furthermore, liver X receptors (LXRs) collaborate with PPARγ in regulating hepatic and adipose lipogenesis during obesity and insulin resistance (**Table 4**).

**Table 4:** Dysregulation of lipid metabolic networks in adipose dysfunction.

Mechanism/Protein	Expression/Activity	Consequences	Metabolic impact	References
PPARγ	Increased	Enhanced adipogenesis	Promotes fat cell differentiation and lipid storage	(187, 188, 191)
SREBP1c	Increased	Increased lipogenesis	Upregulates fatty acid and triglyceride synthesis	(180, 183, 192)
LPL	Dysregulated	Altered fatty acid uptake and storage	Overexpression promotes excessive fat storage; underexpression limits fatty acid utilization	(181, 182, 190)
HSL	Increased	Elevated lipolysis	Increases FFA release from AT, contributing to metabolic disruptions	(19, 35, 36, 181)
ATGL	Increased	Amplified triglyceride breakdown	Converts stored triglycerides to FFAs and glycerol, increasing circulating FFAs	(19, 34, 184)
CD36	Increased	Enhanced fatty acid transport	Facilitates cellular fatty acid uptake and contributes to ectopic fat deposition in non-ATs	(182, 190)
FABP4	Increased	Improved fatty acid trafficking	Supports intracellular fatty acid transport but excessive levels are linked to insulin resistance and inflammation	(180, 183, 190)
Circulating FFAs	Elevated	Metabolic disruption	Induces insulin resistance, hepatic steatosis, and lipotoxicity in non-ATs	(4, 8)
Perilipin	Disrupted	Impaired lipid storage regulation	Disruption leads to unregulated lipolysis and FFA spillover, contributing to metabolic dysfunction	(185, 186, 189)

Summary of key regulatory proteins and pathways governing lipid homeostasis. The table catalogs expression patterns and activity states of central metabolic regulators (PPARγ, SREBP1c), lipolytic enzymes (HSL, ATGL), lipid transport proteins (CD36, FABP4, LPL), and structural components (perilipin). Molecular consequences of altered protein function are mapped to specific metabolic outcomes. Emphasis is placed on the integration of these pathways with mitochondrial energetics and their collective impact on systemic metabolism. Downstream effects on tissue lipid distribution, insulin sensitivity, and hepatic lipid accumulation are detailed. The table illustrates how perturbations in these interconnected pathways drive progressive metabolic dysfunction and lipotoxicity. PPARγ, Peroxisome Proliferator-Activated Receptor Gamma; SREBP1c, Sterol Regulatory Element-Binding Protein 1c; LPL, Lipoprotein Lipase; HSL, Hormone-Sensitive Lipase; ATGL, Adipose Triglyceride Lipase; CD36, Cluster of Differentiation 36; FABP4, Fatty Acid Binding Protein 4; FFAs, Free Fatty Acids.

### 4.7 Metabolic regulation through protein farnesylation in AT

The post-translational addition of farnesyl groups to proteins plays a key role in AT metabolic regulation. Insulin triggers farnesyltransferase activity in adipocytes, leading to p21Ras modification and subsequent Mitogen-activated protein kinase (MAPK) pathway activation - a crucial sequence for metabolic signaling (193). Members of the Ras GTPase family exhibit distinct requirements for this lipid modification: H-Ras localizes exclusively to lipid rafts, while K-Ras4B shows plasma membrane preference through its polybasic domain. Data from adipocyte culture systems demonstrate insulin-stimulated Ras farnesylation drives both adipogenic differentiation programs and glucose transport mechanisms through extracellular signal-regulated kinase 1/2 (ERK1/2) activation (194, 195). Recent work has uncovered parallel MAPK activation pathways operating through farnesylation-independent mechanisms in mature adipocytes, highlighting the complexity of these signaling networks (196).

The Rho GTPase family undergoes similar prenyl modifications, though through more complex regulatory networks affecting glucose metabolism (197, 198). RhoA, Rac1, and Cdc42 require carefully balanced prenylation - both farnesyl and geranylgeranyl additions prove necessary for proper membrane targeting and effector interactions. Insulin signaling promotes Rab protein geranylgeranylation, particularly Rab4 and Rab11, facilitating GLUT4 vesicular trafficking in AT (173). Disrupting these modifications through statins or specific prenylation inhibitors blocks preadipocyte differentiation through impaired cytoskeletal remodeling (199) and compromises glucose-stimulated insulin release from pancreatic β-cells (200).

SREBP transcription factors, particularly SREBP-2, coordinate mevalonate pathway flux to generate prenylation substrates farnesyl pyrophosphate (FPP) and geranylgeranyl pyrophosphate (GGPP) through regulated intramembrane proteolysis (198, 201, 202). These regulatory proteins undergo cholesterol-dependent processing in the Golgi, releasing active nuclear forms that control both isoprenoid and fatty acid synthesis. SREBP-2 preferentially activates genes involved in cholesterol biosynthesis while modulating fatty acid synthesis through FPP-dependent mechanisms. Complex feedback loops connect SREBP activity to cellular sterol levels, prenylation substrate availability, and lipid metabolism through LXR-dependent pathways (201, 202).

Experimental manipulation of protein farnesylation reveals its broad metabolic impact. Loss of normal farnesylation disrupts IR trafficking dynamics and surface expression patterns through altered endosomal sorting mechanisms, contributing to cellular insulin resistance (203). The farnesylation machinery also influences GLUT4 movement through effects on cytoskeletal organization and membrane microdomain composition (174). Within pancreatic β-cells, farnesylation-dependent Raf/ERK signaling couples glucose sensing to insulin secretion through K-ATP channel regulation (204). These findings establish protein farnesylation as a central coordinator of AT function, with dysregulation leading to metabolic disease manifestations including impaired glucose uptake, altered lipid storage, and disrupted adipokine secretion (**Table 5**).

**Table 5:** Protein farnesylation networks in adipose metabolism and disease.

Mechanism/Protein	Expression/Activity	Consequences	Metabolic impact on AT	Regulatory influence	References
Farnesyltransferase (FTase)	Stimulated by insulin	Enhances farnesylation and activation of p21Ras	Critical for insulin signaling and adipocyte function	Disruption can lead to insulin resistance and metabolic disorders	(193–196)
Ras family (H-Ras, N-Ras, K-Ras)	Undergo farnesylation	Essential for membrane localization and activation	Important for insulin-induced adipocyte differentiation and glucose uptake	Enhanced by insulin stimulation	(193–195)
Rho family of GTPases	Undergo prenylation	Vital for glucose homeostasis and metabolic regulation	Balance between farnesylation and geranylgeranylation is crucial	Disruption can lead to pathological conditions	(197–199)
Rab proteins	Geranylgeranylation promoted by insulin	Involved in vesicle trafficking	Impairment affects insulin-induced preadipocyte differentiation	Prenylation inhibition disrupts insulin secretion in pancreatic β-cells	(173, 174, 200)
SREBPs	Regulate mevalonate pathway	Produce FPP and geranylgeranyl pyrophosphate (GGPP)	Influence fatty acid synthesis	Activation influenced by cellular lipid levels	(197, 201, 202)
IR	Affected by farnesylation	Impaired trafficking and recycling	Contributes to insulin resistance	Disruption leads to metabolic dysfunction	(203–205)
GLUT4	Translocation affected by farnesylation	Involved in glucose uptake	Critical for glucose metabolism	Dependent on farnesylation for proper function	(174, 194, 195, 204)
Raf/ERK pathway	Dependent on farnesylation	Involved in glucose-induced insulin secretion	Essential for metabolic regulation	Disruption affects insulin secretion	(194–196, 204)

Experimental evidence linking protein farnesylation to AT function and metabolic disease progression. Key molecular pathways emerge from insulin-activated farnesyltransferase signaling, particularly through GTPase modifications. Detailed analysis reveals interconnected regulatory circuits - from p21Ras activation to downstream metabolic effectors. The data encompasses both physiological and pathological states, mapping how disrupted farnesylation triggers metabolic dysfunction. Evidence spans multiple molecular families including Ras/Rho GTPases, Rab trafficking proteins, and SREBP transcriptional networks. Experimental findings highlight critical roles in IR dynamics, glucose transport through GLUT4, and Raf/ERK signal propagation. These molecular interactions provide mechanistic insights into how altered protein farnesylation drives metabolic disease development. FTase, Farnesyltransferase; H-Ras, N-Ras, K-RasRas family, Rho family of GTPases, Rab proteins; SREBPs, Sterol Regulatory Element-Binding Proteins; FPP, Farnesyl Pyrophosphate; GGPP, Geranylgeranyl Pyrophosphate; IR, Insulin Receptor; GLUT4, Glucose Transporter Type 4; Raf/ERK, Raf/Extracellular Signal-Regulated Kinase pathway.

### 4.8 Lipid metabolism and trafficking

Disruption of normal lipid storage patterns frequently leads to accumulation within non-adipose tissues (non-ATs). This abnormal fat deposition triggers pathological cascades in cardiovascular and metabolic systems. The resulting tissue damage involves multiple molecular mechanisms (205, 206). Intracellular lipid homeostasis depends on precise transcriptional regulation. Recent studies have established the carbohydrate response element-binding protein (ChREBP) as essential in this process (192). The enzyme networks controlled by ChREBP regulate cellular lipid synthesis. Key components include fatty acid synthase (FAS), which generates palmitate molecules. Acetyl-CoA carboxylase (ACC) supplies critical malonyl-CoA building blocks. Stearoyl-CoA desaturase-1 (SCD1) creates specific double bonds in fatty acid chains (207). Each enzyme performs distinct catalytic roles in lipid metabolism. Additionally, emerging evidence suggests that circadian regulation of these enzymes significantly impacts their activity patterns (208).

In vulnerable tissues - particularly liver parenchyma, striated muscle, and myocardium - excess lipid burden initiates both cell death pathways and inflammatory responses (209). The buildup of specific lipid species, especially Diacylglycerol (DAGs) and ceramides, causes oxidative damage through ROS generation. These changes trigger progressive mitochondrial dysfunction (210). Impaired respiratory chain activity further increases ROS production. Local tissue inflammation worsens. A self-reinforcing cycle of metabolic deterioration emerges. Recent work has identified the endocannabinoid system (ECS) as a key mediator in lipotoxicity, with Cannabinoid receptor type 1 (CB1) activation promoting lipogenesis and inflammation (211). The newly characterized role of mitochondrial-associated membranes (MAMs) in lipid trafficking adds another layer of complexity to this pathological cascade. MAMs regulate lipid metabolism, calcium homeostasis, and ROS generation (212). Disruption of MAM integrity can lead to increased ROS production, mitochondrial damage, and activation of inflammatory pathways (213).

The effects manifest differently across organ systems. Hepatocytes develop steatosis and advance toward non-alcoholic fatty liver disease (NAFLD). Skeletal muscle fibers show severe insulin resistance. Cardiac function deteriorates. Pancreatic β-cells display significant secretory deficits (214, 215). Despite tissue-specific manifestations, common pathogenic mechanisms exist: disrupted AT function floods circulation with FFAs, overwhelming normal lipid processing pathways (215, 216). The resulting cellular stress responses - from ER protein folding defects to mitochondrial dysfunction - amplify metabolic disruption. Recent research highlights the crucial role of EVs in inter-organ metabolic communication and disease progression. EVs, including exosomes, mediate intercellular and inter-organ crosstalk by carrying bioactive molecules such as proteins, lipids, and microRNA (miRNAs) (217). These mechanistic insights into ectopic fat accumulation highlight several therapeutic targets. Pharmacological regulation of lipogenic enzymes, enhancement of FA oxidation, and suppression of inflammatory mediators could interrupt disease progression. The increasing prevalence of these disorders necessitates rapid therapeutic development. Success will likely require concurrent targeting of both cellular stress responses and systemic metabolic dysfunction.

### 4.9 Environmental obesogens and their impact on adipogenic regulation

Environmental obesogens represent endocrine-disrupting chemicals that promote obesity through alterations in adipogenesis and metabolic homeostasis (218, 219). Tributyltin (TBT), a well-characterized obesogen, activates retinoid X receptor (RXR) and PPARγ-key transcriptional regulators of adipocyte differentiation (218, 220). Critical developmental exposure to TBT enhances adipocyte differentiation, modifies gene expression profiles, and induces persistent obesogenic phenotypes (218, 221). The spectrum of identified obesogens encompasses bisphenol A, phthalates, and perfluorinated compounds (219).

The obesogen hypothesis proposes that prenatal exposure reprograms stem cells toward preferential adipocyte differentiation, potentially establishing transgenerational inheritance patterns (222). Recent investigations demonstrate that developmental obesogen exposure increases AT formation and fat storage capacity in offspring, with effects persisting across generations (223, 224). These compounds, prevalent in pesticides, food packaging, and cosmetics, reprogram adipose stem cells through epigenetic mechanisms including DNA methylation alterations and chromatin remodeling (225–228). Transgenerational consequences include increased white adipose depot weights, adipocyte hyperplasia and hypertrophy, and hepatic lipid accumulation (223, 229).

Population biomonitoring reveals widespread exposure to bisphenol A (BPA) and its structural analogues-bisphenol S (BPS) and bisphenol F (BPF)-detected in consumer products and biological fluids (230–233). These substitutes exhibit comparable or enhanced endocrine-disrupting potential relative to BPA (232). *In vitro*studies demonstrate bisphenol-mediated promotion of adipogenesis and lipid accumulation in human adipocytes through interference with adipogenic gene expression and metabolic pathways (234–236).

Epidemiological investigations establish significant associations between BPA exposure and metabolic perturbations. Meta-analyses report increased obesity odds ratios of 1.40-1.76 correlating with elevated BPA concentrations (237–240). BPA exposure correlates with abdominal obesity risk (odds ratios: 1.31-1.62) and increased BMI and waist circumference (239–242). Mechanistic evidence suggests BPA functions as an obesogen through hormonal receptor modulation and metabolic syndrome promotion (243). However, cross-sectional study designs and single-point measurements limit causal inference capabilities (244).

Emerging evidence indicates BPA alternatives, including Bisphenol AF (BPAF), demonstrate similar endocrine-disrupting profiles associated with obesity, glucose dysregulation, and cardiovascular abnormalities (245, 246). Mechanistic investigations reveal these compounds activate PPARγ pathways, stimulate adipocyte hypertrophy, and dysregulate adipogenic networks (234, 236). Exposure induces lipid accumulation, pro-inflammatory cytokine expression, and impaired insulin signaling in human adipocytes (235, 247, 248). BPAF specifically compromises mitochondrial function and promotes adipose inflammation (249).

Current research priorities encompass: identifying adipose-specific molecular targets through single-cell genomics; characterizing critical developmental windows via longitudinal cohorts; and elucidating transgenerational effects using multi-generational models. Advanced analytical platforms reveal novel obesogenic compounds, necessitating regulatory reassessment. Intervention strategies under investigation include targeted nutritional approaches and exposure reduction protocols. Understanding gene-environment interactions, particularly metabolic gene polymorphisms, may facilitate personalized prevention strategies for preserving AT function.

### 4.10 Lifestyle interventions and environmental factors in AT function

AT plays a crucial role in regulating whole-body metabolism and energy homeostasis (250). Exercise and physical activity significantly impact AT function through multiple mechanisms, including enhanced mitochondrial biogenesis, increased oxidative capacity, and reduced inflammation (251, 252). Regular exercise promotes AT remodeling, improves metabolic flexibility, and stimulates the browning of WAT (251, 253). These effects contribute to improved insulin sensitivity and reduced risk of cardiometabolic disorders (254). AT dysfunction, often associated with obesity and aging, can lead to various metabolic disorders (3). However, exercise-induced changes in adipokine secretion and lipid composition can positively influence other organs and tissues, promoting overall metabolic health (255, 256).

Exercise-induced browning of WAT has emerged as a promising mechanism for improving metabolic health. Physical activity stimulates the release of myokines like irisin and FGF21 from skeletal muscle, which promote WAT browning (257–259). This process involves increased expression of UCP1 and enhanced mitochondrial function, leading to improved thermogenesis and energy expenditure. High-intensity exercise appears more effective in inducing WAT browning compared to low-intensity exercise (260). The browning effect is mediated through various pathways, including β-adrenergic signaling, ROS, and exerkines (261). Irisin, in particular, plays a crucial role by binding to integrin αV/β5 receptors and promoting WAT browning (262). Furthermore, irisin supplementation or exercise-induced irisin activation may offer therapeutic potential for metabolic disorders (263).

Dietary interventions beyond caloric restriction can significantly impact AT function and inflammation. The Mediterranean diet, rich in monounsaturated fats and polyphenols, reduces adipose inflammation by suppressing NF-κB and MAPK pathways while activating AMPK (264, 265). Intermittent fasting and ketogenic diets improve mitochondrial function, reduce inflammation, and enhance autophagy in AT (266, 267). These diets modulate gut microbiota composition, decreasing lipopolysaccharide-producing bacteria and inflammatory signaling in monocytes (266). Caloric restriction and low-fat diets both promote weight loss and reduce macrophage infiltration in AT, with caloric restriction showing superior effects on mitochondrial metabolism (268). The Mediterranean diet supplemented with almonds improves AT biology by promoting angiogenesis, adipogenesis, and M2-like macrophage polarization (269). These dietary interventions offer promising strategies for managing obesity-related inflammation and metabolic dysfunction.

Environmental factors, particularly cigarette smoking, significantly impact AT homeostasis and function. Smoking induces AT dysfunction through multiple pathways, including increased lipolysis, inflammation, and insulin resistance (270, 271). Nicotine activates AMPKα2 in adipocytes, leading to increased lipolysis and free fatty acid release (271). Smoke-derived oxidants promote adipose inflammation by recruiting macrophages and increasing pro-inflammatory cytokine production (272, 273). This chronic low-grade inflammation disrupts insulin signaling, contributing to insulin resistance. Smoking-induced adipose dysfunction is characterized by altered adipocyte differentiation, impaired insulin action, and dysregulated adipokine secretion (274). These effects extend beyond AT, impacting whole-body metabolism and increasing the risk of various metabolic disorders.

Chronic alcohol consumption and obesity significantly disrupt AT homeostasis, leading to fibrosis and metabolic dysfunction. Alcohol impairs adipocyte differentiation, reduces adiponectin expression, and promotes inflammation. It also increases lipolysis and ectopic fat deposition, contributing to fatty liver disease (275). Obesity-induced AT fibrosis involves complex cellular interplays, including macrophage infiltration and preadipocyte activation (276, 277). The Hippo pathway, in conjunction with Transforming growth factor- β (TGF-β) signaling, plays a crucial role in adipocyte plasticity and fibrosis development (278). TGF-β superfamily members regulate adipocyte differentiation, fibrosis, and metabolic functions (279). Autophagy dysregulation in AT may contribute to alcohol-induced liver injury (280).

Sleep deprivation significantly impacts AT function and adipokine secretion patterns. Reduced sleep duration is associated with increased leptin and visfatin levels, potentially contributing to inflammation and insulin resistance (281). Sleep loss also decreases adiponectin levels, which may lead to metabolic dysregulation (282). The circadian clock plays a crucial role in regulating adipokine secretion, as demonstrated by the blunted metabolic response to sleep restriction in Per1/2 mutant mice (283). Interestingly, chronic sleep deprivation is associated with higher adiponectin levels in patients with endocrine metabolic disorders, possibly as a compensatory mechanism (284). The day/night pattern of leptin is influenced by both the endogenous circadian pacemaker and behavioral factors such as sleep and food intake (285).

The mechanisms underlying metabolic dysfunction in AT extend beyond cellular senescence to include multiple interconnected pathways. Chronic overnutrition leads to adipocyte hypertrophy and tissue hypoxia, while persistent psychological stress activates glucocorticoid signaling and promotes visceral fat accumulation. Circadian rhythm disruption alters adipose metabolic gene expression patterns, affecting normal metabolic oscillations. Environmental pollutant exposure (as discussed in section 4.9) contributes to adipose dysfunction through multiple mechanisms. Additionally, gut microbiome dysbiosis influences adipose inflammation through altered production of short-chain fatty acids, establishing a complex gut-adipose axis in metabolic regulation.

Understanding these modifiable factors provides critical opportunities for preventing and treating adipose dysfunction through comprehensive lifestyle interventions rather than relying solely on pharmacological approaches. Future research should focus on personalized lifestyle prescriptions based on individual AT characteristics and metabolic phenotypes.

### 4.11 Regulatory networks of small RNAs in adipose metabolism

MicroRNA-mediated regulation of gene expression occurs through binding to partially complementary sequences primarily in the 3’ untranslated regions (3’ UTRs) of target messenger RNAs, though binding can also occur in 5’ UTRs and coding regions. This interaction typically leads to translational repression and/or mRNA decay, with the relative contribution of each mechanism varying by cellular context and the degree of complementarity. The regulatory influence of miRNAs spans multiple cellular pathways, with current bioinformatic predictions and experimental evidence suggesting that miRNAs may regulate the majority (estimated 60-90%) of mammalian protein-coding genes through complex regulatory networks (286–288).

AT function depends on precise microRNA-mediated regulation. Distinct microRNA populations control white adipocyte differentiation and brown/beige adipocyte thermogenic programming (289, 290). The impact of these regulatory RNAs extends to metabolic tissues including pancreatic β-cells, hepatocytes, and skeletal muscle (291). High-throughput sequencing has revealed tissue-specific expression patterns that coordinate systemic metabolic responses (292).

The let-7 family highlights the complexity of microRNA-mediated metabolic control. These regulators target key components of glucose homeostasis and insulin signaling networks, with their expression significantly diminished in diabetic tissues (293, 294). Obesity alters microRNA profiles across metabolic organs, though these changes can normalize following weight reduction (295, 296). Notably, adiponectin regulation involves miR-193b activity, linking obesity-associated decreases in this microRNA to broader metabolic dysfunction (297, 298).

Analysis of AT from obese subjects reveals characteristic alterations in microRNA expression. Fat depot expansion correlates with increased miR-221 and altered patterns of miR-17-5p and miR-132 across anatomical locations (299–301). Detection of these molecules in blood points to their role in systemic metabolic regulation (302, 303). MiR-223 exhibits key functions in metabolic homeostasis. Its abundance increases in subcutaneous fat during insulin resistance development (304) and shapes inflammatory responses in tissue macrophages (305, 306). Notably, circulating miR-223 decreases as obesity progresses toward T2DM (307).

The miR-130 family members suppress adipogenesis through PPARγ inhibition (308) and mediate inflammatory signaling (309). Their reduced expression in obese subcutaneous AT (310) affects inflammatory responses and insulin sensitivity through altered immune cell function, with implications for metabolic syndrome progression (292, 302).

Recent advances highlight emerging regulatory mechanisms in adipose dysfunction. Epigenetic modifications play a crucial role in regulating stem cell fate and function, particularly in adipose-derived stem cells (ADSCs). DNA methylation, histone modifications, and chromatin remodeling fundamentally reprogram cell fate decisions and metabolic capacity (311, 312). These epigenetic mechanisms influence ADSC differentiation into various lineages, including osteogenic and adipogenic pathways (313). In the context of obesity and type 2 diabetes, DNA methylation events are associated with altered AT function and gene regulation (314, 315). Novel signaling crosstalk between AMP-activated protein kinase (AMPK) and TBC1 domain family member ¼ (TBC1D1) reveals additional layers of insulin-independent glucose uptake regulation (316), while tissue-specific microRNA networks, particularly miR-223 and miR-130, regulate complex inflammatory and metabolic responses through post-transcriptional control (310, 317, 318). These emerging pathways provide new therapeutic targets beyond traditional approaches.

### 4.12 Disrupted adipogenesis in metabolic disease

Transcription of adipogenic genes begins with chromatin binding of CCAAT/enhancer-binding protein (C/EBPβ) at target promoters. DNA binding sites for PPARγ and C/EBPα become accessible during subsequent phases, allowing transcriptional activation of differentiation factors. DNA accessibility changes through histone modifications at adipogenic gene promoters. The differentiation program incorporates additional layers of control through methylation patterns, chromatin structure alterations, and specific microRNA expression. Transcriptional networks coordinate with chromatin remodeling complexes to establish cell-type specific gene expression patterns (319–321).

Bone morphogenetic protein signaling activates early commitment factors in mesenchymal precursor cells. Wnt pathway activation modifies chromatin structure at adipogenic loci, enabling progression toward the adipocyte phenotype. Further maturation yields functional fat cells containing characteristic lipid stores and metabolic enzymes. Disruption of these molecular pathways impairs AT development and function, leading to systemic metabolic deterioration. The transition between developmental stages requires precise temporal control of multiple signaling cascades. Defects in these regulatory networks prevent proper adipocyte maturation (322, 323).

### 4.13 ECM remodeling

#### 4.13.1 Matrix composition changes

AT matrix undergoes substantial reorganization during obesity, marked by elevated deposition of fibrillar collagens and advancing fibrosis. Analysis of matrix composition reveals that increased collagen VI levels regulate both metabolic function and inflammatory states. Molecular studies show collagen VI deposition initiates cellular responses including enhanced inflammatory mediator production and disrupted insulin signaling networks (97). Proteomic profiling reveals elevated expression of multiple matrix metalloproteinase (MMPs), including MMP-2, -3, -12, -14, -19, alongside increased TIMP-1 levels within obese adipose samples, reflecting dynamic matrix restructuring (81). While obesity shifts MMP/TIMP (tissue inhibitor of metalloproteinases) ratios toward enhanced degradation, this compensatory response fails to prevent progressive fibrosis (82). Multiple matrix components including distinct collagen types, fibronectin molecules, and hyaluronan networks interact with cellular receptors such as integrins and Cluster of differentiation 44 (CD44), activating signaling cascades that regulate cellular metabolism and inflammatory pathways (324). Research demonstrates that adipocyte differentiation requires coordinated matrix remodeling, as experimental MMP inhibition disrupts normal adipogenesis (82). Detailed characterization of matrix regulation pathways (**Table 6**) suggests therapeutic opportunities through targeted modification of specific matrix components and their regulatory enzymes.

**Table 6:** Molecular architecture of matrix remodeling in obese AT.

Regulatory component	Expression pattern	Molecular function	Metabolic impact	References
ECM Structural Components				
Fibrillar Collagens	Increased 2–3 fold in obesity	• Forms rigid scaffold structure • Modifies tissue mechanics • Alters mechanotransduction	• Limits adipocyte expandability • Promotes inflammatory signaling • Impairs metabolic flexibility	(81, 325, 326)
Collagen VI	2–3 fold upregulation in obese AT	• Modifies pericellular matrix • Activates inflammatory pathways • Alters mechanical properties	• Enhances fibrosis • Promotes insulin resistance • Impairs adipogenesis	(81, 83, 326)
Mechanical Sensors				
YAP/TAZ Complex	Activated by matrix stiffening	• Responds to mechanical stress • Controls fibrotic gene expression • Regulates adipocyte function	• Promotes fibrosis • Alters adipocyte differentiation • Modifies metabolic function	(325, 327–329)
Matrix-Modifying Enzymes				
MMPs (2, 3, 12, 14, 19)	2–5 fold increase in obesity	• Degrades ECM components • Regulates matrix turnover • Releases bioactive factors	• Modifies tissue architecture • Influences adipogenesis • Affects metabolic function	(81, 82)
TIMP-1	2–3 fold elevation in obesity	• Inhibits MMP activity • Controls matrix turnover • Regulates tissue remodeling	• Promotes matrix accumulation • Contributes to fibrosis • Affects metabolic health	(81, 83, 330)
Metabolic Regulators				
AMPK	Decreased in obesity	• Suppresses fibrosis • Regulates metabolism • Controls inflammation	• Improves insulin sensitivity • Reduces inflammation • Maintains ECM homeostasis	(331–333)
Microenvironment Modifiers				
HIF-1α	Increased in obese AT	• Responds to hypoxia • Induces fibrotic response	• Promotes matrix accumulation • Enhances inflammation • Impairs metabolic function	(81, 83, 325)
Cell Surface Receptors				
Integrins/CD44	Altered expression in obesity	• Sense matrix properties • Transduce mechanical signals • Mediate cell-ECM interaction	• Affect cellular metabolism • Influence inflammation • Modify tissue function	(325, 326, 334)

This table systematically categorizes the key molecular components regulating AT matrix remodeling during obesity. Components are organized by functional categories (structural, mechanical sensors, modifying enzymes, metabolic regulators, microenvironment modifiers, and cell surface receptors), with quantitative changes in expression/activity provided where documented in research. The analysis reveals coordinated regulation between matrix structural elements, their modifying enzymes, and associated signaling pathways. Notably, obesity-associated changes in these pathways (2–5 fold changes in key regulators) create interconnected feedback loops affecting tissue architecture, inflammatory status, and metabolic function. Understanding these relationships is crucial for developing targeted therapeutic strategies for obesity-related metabolic dysfunction. Each component’s molecular functions and metabolic impacts are presented to demonstrate how local tissue remodeling influences systemic metabolic health. ECM, Extracellular Matrix; YAP/TAZ, Yes-Associated Protein/Transcriptional Coactivator with PDZ-binding motif Complex; MMPs, Matrix Metalloproteinases; TIMP-1, Tissue Inhibitor of Metalloproteinases-1; AMPK, AMP-Activated Protein Kinase; HIF-1α, Hypoxia-Inducible Factor 1-Alpha; Integrins/CD44, Integrins/Cluster of Differentiation 44.

#### 4.13.2 Mechanical stress

Mechanical testing demonstrates increased tissue stiffness that limits adipocyte expansion and triggers mechanotransduction through focal adhesion complexes (327, 328). Research demonstrates that adipocytes sense elevated matrix rigidity through mechanosensitive pathways, resulting in increased pro-fibrotic gene transcription and accelerated matrix protein synthesis. These mechanical signals operate via organized actin cytoskeletal networks and mechanosensitive transcription factors, particularly the (Yes-associated protein/Transcriptional coactivator with PDZ-binding motif (YAP/TAZ) complex (329). Expanding adipose depots develop localized hypoxia that activates hypoxia-inducible factor 1- α (HIF-1α)-dependent signaling cascades, establishing self-perpetuating cycles of matrix accumulation. These matrix modifications propagate beyond AT to influence systemic metabolism, demonstrating the central role of matrix remodeling in obesity pathophysiology (325).

#### 4.13.3 Fibrosis development

Matrix protein dynamics emerge as key determinants of AT plasticity during metabolic disease progression. Studies reveal that obesity-driven matrix deposition creates fibrotic microenvironments that sustain inflammatory responses and compromise insulin signaling pathways (83). Analysis shows that metformin modulates metabolism through AMPK activation across multiple tissues, though the precise mechanisms linking AMPK activation to improved insulin sensitivity remain an area of active investigation (331). Metformin treatment actively suppresses matrix buildup and fat tissue scarring that typically accompany obesity-driven insulin resistance, operating through multiple cellular mechanisms. At the molecular level, metformin triggers AMPK activation, which interferes with TGF-β1/Smad3 signaling - a key driver of tissue fibrosis. This interference reduces collagen formation and dials down genes involved in the scarring process (332). Metformin also modulates other critical pathways, dampening integrin/ERK signaling, limiting matrix-degrading enzymes, and protecting enlarged fat cells from premature death (334). These molecular mechanisms help maintain appropriate matrix elasticity during tissue expansion (328). Additionally, AMPK pathway activation in AT suppresses inflammatory signaling networks while improving insulin sensitivity (333). The demonstrated effects of metformin on both AMPK signaling and matrix remodeling provide multiple therapeutic targets for treating obesity-related metabolic disorders.

### 4.14 The role of autophagy in AT function and metabolic disorders

Autophagy maintains AT homeostasis by regulating adipocyte development, metabolism, and inflammatory status. This cellular recycling pathway removes damaged proteins and organelles, supporting proper cell function. Impaired autophagy in AT drives metabolic disease. Studies link autophagy defects to obesity and insulin resistance. The progression to T2DM accelerates when autophagy fails (335–337).

Autophagy regulates adipose function through distinct mechanisms. It controls lipid droplets via lipophagy pathways. The process degrades specific proteins to modulate adipokine release. Mitochondrial quality depends on mitophagy-mediated turnover. Mouse models reveal the metabolic impact of autophagy. Adipose-specific deletion of Atg7 reduces white fat mass. These mice show poor adipogenesis and whole-body insulin resistance (338, 339).

Obese AT exhibits heightened autophagy markers, yet suppressing this pathway yields metabolic benefits (340). While obesity initially triggers increased autophagy as an adaptive response, chronic metabolic stress leads to autophagy dysfunction. Single-cell transcriptomics and proteomics studies reveal that suppressing excessive autophagy in established obesity paradoxically improves metabolic outcomes by reducing inflammatory activation. The autophagic machinery influences adipocyte phenotype transitions between white and brown states, affecting whole-body energy balance (341). Understanding autophagy regulation in mature fat cells remains incomplete, highlighting the need for mechanistic studies to guide therapy development.

Defective autophagy pathways alter adipocyte differentiation, lipid handling, and insulin responses (335). Body fat distribution patterns, especially visceral depot expansion, compound these metabolic perturbations. This inflammatory state within AT raises the likelihood of developing metabolic diseases, including insulin resistance and cardiovascular problems (342). The autophagic machinery helps regulate immune responses at multiple levels - from bacterial clearance to immune cell activation and inflammatory mediator production (343). When autophagy fails, inappropriate inflammatory activation accelerates disease progression (344). Sustained inflammation suppresses autophagy function, establishing self-perpetuating pathological cycles (345). In acute kidney injury models, autophagy attenuates inflammatory damage via mTOR and AMPK signaling networks (346). Clinical intervention strategies targeting autophagy - through dietary modification, exercise programs, and drug development - aim to restore AT health and metabolic function.

### 4.15 Metabolic regulation by adipose-derived extracellular vesicles

Secretion of extracellular vesicles (EVs) from WAT represents a key mechanism in metabolic regulation between organs (347, 348). Analysis of vesicular content has identified specific metabolic enzymes and adipose hormones, alongside regulatory RNAs that influence target cell signaling pathways (349, 350).

Electron microscopy coupled with nanoparticle tracking analysis distinguishes vesicle subpopulations through unique biophysical properties and specific protein markers. Endosomal sorting complex required for transport (ESCRT)-dependent exosome formation generates 30–150 nm particles characterized by CD63, CD81, and tumor susceptibility gene 101 (TSG101) expression. Calcium-dependent membrane budding produces larger 100–1000 nm microvesicles expressing annexin V and selectins. Membrane phospholipid redistribution during apoptosis yields >1000 nm apoptotic bodies marked by phosphatidylserine externalization (351). Each population exhibits distinct membrane protein topology and internal cargo composition, allowing targeted isolation and characterization. These distinct vesicle populations serve unique roles in intercellular communication and metabolic regulation.

The vesicular miRNA cargo plays a crucial role in metabolic regulation. Studies investigating vesicular RNA content have demonstrated functional consequences in metabolic tissues. Through direct modulation of glucose transporter expression, miR-222 regulates skeletal muscle metabolism, concurrent with miR-23b-mediated effects on insulin signaling proteins (350, 352). The resulting perturbations in glucose homeostasis and hepatic lipid handling establish a mechanistic framework for vesicle-mediated metabolic regulation (350, 353).

Obesity alters both the protein composition and signaling effects of adipose-derived vesicles. Mass spectrometry has identified increased inflammatory mediators and decreased adiponectin levels, along with changes in lipid transport proteins (351, 354). These alterations promote inflammatory responses in AT and disrupt glucose homeostasis in liver and muscle (348). The dysregulation of vesicle secretion and composition represents a fundamental mechanism linking obesity to systemic metabolic dysfunction.

Pathological changes in adipocytes influence vesicle composition through specific molecular mechanisms (351). Hyperglycemia-stressed adipocytes release vesicles enriched in pro-inflammatory miRNAs that activate macrophage responses and tissue inflammation. These vesicles interact with cellular targets through EphB2-ephrinB1 binding, affecting insulin signaling and lipid trafficking pathways (350). BAT vesicles show unique properties through miR-92a and BMP7 enrichment, supporting thermogenic programming and metabolic balance (355). The differential regulation of vesicle production and content between white and BAT highlights their specialized roles in metabolic control.

The molecular characteristics of adipose-derived vesicles enable their use as diagnostic tools and therapeutic vectors in metabolic disease management (356). Their composition serves as biomarkers for metabolic syndrome, cardiovascular disease, and cancer progression (357). Clinical applications include longitudinal disease monitoring and therapeutic response assessment (358). Current research focuses on engineering vesicles for targeted drug delivery and metabolic modulation through cargo manipulation. These tissue-specific modifications provide mechanistic insights while suggesting novel therapeutic approaches for metabolic disorders. The development of standardized isolation protocols and stability enhancement methods addresses key challenges in clinical translation.

## 5 Systemic impact of adipose dysfunction

AT dysfunction serves as the primary driver of systemic metabolic dysregulation, orchestrating metabolic perturbations across liver, skeletal muscle, pancreas, and other organs through integrated molecular mechanisms. The following sections detail how compromised AT propagates dysfunction through altered adipokine secretion, excessive free fatty acid release, inflammatory mediator production, and EV signaling, establishing self-perpetuating cycles that progressively worsen whole-body metabolic homeostasis.

### 5.1 Endocrine disruption

#### 5.1.1 Adipokine dysregulation

Beyond storing energy, AT actively produces proteins called adipokines. These factors act throughout the body, profoundly affecting metabolism, immune function, and insulin signaling pathways (359, 360). Within healthy AT, specific adipokines work together to regulate appetite, energy use, and tissue responses to insulin (361). AT produces several metabolically critical proteins, including the energy-regulating hormone leptin and the insulin-sensitizing factor adiponectin. Additional secreted factors like resistin, visfatin, and retinol binding protein 4 (RBP4) also shape metabolic outcomes. Targeted gene deletions in mouse models highlight their unique functions - leptin knockouts develop severe obesity with decreased energy expenditure, while mice lacking adiponectin exhibit profound insulin resistance. Similarly, genetic ablation of resistin, visfatin or RBP4 leads to complex changes in inflammatory signaling networks and systemic glucose regulation (361, 362).

Obesity and T2DM trigger pronounced alterations in AT structure and function, disrupting normal adipokine production patterns (363). Recent studies demonstrate depot-specific adipokine secretion patterns, with visceral fat showing distinct inflammatory profiles compared to subcutaneous deposits. These depot-specific differences contribute significantly to metabolic outcomes. The resulting secretory profile shows suppressed levels of metabolically protective factors like adiponectin alongside increased inflammatory mediators TNF-α and IL-6 (2, 250). This adipokine imbalance initiates and maintains chronic low-grade inflammation, progressively impairing insulin signaling across multiple tissue beds (175).

The inflammatory adipokines resistin and visfatin compromise metabolic health through multiple mechanisms: disrupting IR activation, amplifying inflammatory protein production, and perturbing glucose homeostasis (360). These molecular derangements create recurring cycles of metabolic dysfunction and inflammation. Given their central regulatory roles, adipokine pathways represent attractive therapeutic targets for obesity-related disorders. Current therapeutic development focuses on strategies to normalize adipokine profiles through direct pathway modulation or broader improvements in AT function.

#### 5.1.2 Hormone resistance

Adipose dysfunction manifests through cellular defects and altered tissue architecture. Limited formation of new adipocytes leads to pathological expansion of existing fat cells, reducing lipid storage capacity (364). Tissue hypoxia develops alongside reduced blood vessel formation, triggering inflammatory cascades (365). Analysis of subcutaneous fat reveals altered BMP-4 signaling networks and increased gremlin-1 protein levels in hypertrophic obesity (366, 367). These molecular alterations modify adipokine production profiles, enhance lipolytic activity, and maintain chronic inflammatory states (342).

Loss of IR signaling in fat cells triggers lipase activation, with increased hydrolytic activity. The resulting release of stored lipids promotes fat accumulation in nonadipose organs, disrupting metabolic homeostasis. Ectopic lipid deposition in liver, skeletal muscle, and pancreatic tissue progressively impairs systemic glucose regulation and insulin sensitivity (368).

#### 5.1.3 Metabolic consequences

Pro-inflammatory signals block insulin pathways (4), while hypertrophic fat cells show reduced metabolic responses and trigger fat accumulation in other tissues (369). Inflammatory mediators, oxidative damage, and excess lipids suppress adipocyte formation (364). The limitation in adipose expansion drives metabolic disease progression through multiple mechanisms. Macrophages and T lymphocytes within AT suppress formation of new adipocytes while stimulating connective tissue production. The resulting accumulation of collagen and other matrix proteins creates physical constraints on AT growth and cellular differentiation. Local tissue inflammation and matrix remodeling establish a microenvironment that perpetuates dysfunction through impaired adipogenesis (370).

### 5.2 AT receptor networks and metabolic regulation

AT receptor systems form interconnected signaling networks that regulate metabolic responses. Severe metabolic disruption and lipodystrophy emerge when insulin receptor (IR) or insulin-like growth factor 1 receptor (IGF1R) signaling fails, highlighting their central role in adipose development (371). Beyond simple fat storage, AT demonstrates remarkable plasticity through two distinct growth mechanisms. While both hyperplastic and hypertrophic expansion increase adipose mass, new adipocyte formation through hyperplasia appears to preserve metabolic health during obesity (372). AT dysfunction manifests when expansion limits are reached, forcing lipid accumulation in non-ATs and triggering systemic metabolic deterioration (37, 261).

TLR2 and TLR4 expression patterns shift dramatically in obese AT, establishing these pattern recognition receptors as central inflammatory mediators. Beyond pathogen sensing, these receptors respond to elevated fatty acids and other obesity-associated molecular signals (373). Subsequent NF-κB pathway activation drives macrophage recruitment and amplifies inflammatory cytokine production (374). This inflammatory cascade particularly affects visceral fat deposits, which show heightened susceptibility compared to subcutaneous stores.

AT dysfunction orchestrates systemic metabolic perturbations through distinct molecular mechanisms targeting peripheral organs. Dysfunctional adipocytes release excessive FFAs through uncontrolled lipolysis, which directly impair hepatic insulin signaling by inducing diacylglycerol accumulation and protein kinase C (PKC) activation, leading to hepatic steatosis and gluconeogenesis dysregulation (8, 375). In skeletal muscle, these circulating FFAs promote intramyocellular lipid accumulation, disrupting insulin-stimulated glucose uptake through ceramide-mediated inhibition of Akt phosphorylation (376, 377). Moreover, aberrant adipokine secretion profiles, characterized by elevated TNF-α and IL-6 alongside diminished adiponectin, propagate inflammatory signaling that impairs both hepatic and muscle insulin sensitivity (175, 378). Adipose-derived EV carrying specific miRNA signatures (miR-222, miR-23b) further mediate inter-organ communication, directly altering glucose transporter expression in skeletal muscle and lipid metabolism in hepatocytes (350, 352). This molecular crosstalk establishes a self-perpetuating cycle where adipose dysfunction progressively compromises peripheral tissue metabolic homeostasis.

PPARs regulates complex metabolic networks while directing adipocyte differentiation programs (379). Chromatin landscape remodeling by PPARγ proves essential for adipogenesis (380). These nuclear receptors undergo sophisticated post-translational control, with disrupted modifications linked to obesity and metabolic dysfunction (381, 382). Each PPAR subtype serves specialized metabolic functions - PPARα coordinates fatty acid oxidation pathways while PPARβ/δ enhances lipid metabolism (383). Pharmacological modulation of PPAR receptors represents a therapeutic avenue for metabolic disease intervention, though challenges remain in targeting specificity (384). Within AT, complex signaling networks emerge from adipocyte and macrophage interactions, generating localized inflammation marked by specific immune cell populations and altered cytokine profiles (385). The resulting metabolic stress triggers phosphorylation cascades through several pathways. Experimental evidence points to IKK/NF-κB signaling as a central mediator, while PI3K/Akt and MAP kinase activation leads to serine/threonine modifications of insulin receptor substrates (IRSs), ultimately disrupting glucose homeostasis (386). Pattern recognition receptors and inflammasome complexes sustain this inflammatory state (69). Disrupted adipokine secretion patterns and altered lipid handling further compound insulin resistance development.

Anatomically distinct fat deposits display unique receptor expression patterns and metabolic properties. Upper body and visceral deposits correlate with increased metabolic risk, while gluteofemoral fat appears protective (387). These depot-specific differences stem from varied cellular composition, gene regulatory networks, and physiological functions - including distinct steroid receptor profiles, adipokine signatures, and metabolic activities (388, 389). Recent work has expanded this heterogeneity concept to brown, beige, and ectopic fat, with each depot’s molecular profile differently impacting systemic metabolism (390, 391).

### 5.3 Distinct mechanisms of adipocyte elimination in metabolic disease

AT undergoes several forms of cellular elimination under metabolic stress: mitochondrial-dependent apoptotic pathways, inflammatory cell death via receptor-interacting serine/threonine-protein kinase 3 (RIPK3)/Mixed lineage kinase domain-like protein (MLKL) signaling, and inflammasome-triggered membrane disruption through specific molecular cascades (84). High-resolution microscopy demonstrates characteristic morphological changes: distended ER, damaged mitochondrial networks, and oxidative modifications within hypertrophied adipocytes. These conditions promote NLRP3 inflammasome assembly, triggering caspase-1 activation and subsequent plasma membrane permeabilization (85). Infiltrating macrophages organize around dying adipocytes, forming characteristic inflammatory structures termed crown-like structures (CLS) within affected regions (86). During cell death, released cellular components - including DNA fragments, bioactive lipids, and pro-inflammatory mediators - amplify both local and systemic metabolic disruption through well-defined pathways (392). Adipocyte survival regulation involves intricate crosstalk between death receptor signaling and mitochondrial pathways.

The consequences of adipocyte death extend beyond local tissue dysfunction, driving organ-specific metabolic disruptions through precisely characterized molecular pathways. Released cellular components from dying adipocytes, including mitochondrial DNA fragments and oxidized lipids, trigger hepatic Kupffer cell activation via TLR9 signaling, promoting liver inflammation and fibrosis (393). In skeletal muscle, these damage-associated molecular patterns activate resident macrophages, inducing myocyte insulin resistance through paracrine IL-1β secretion (394). Furthermore, the compensatory hyperinsulinemia resulting from adipose dysfunction stimulates *de novo*lipogenesis in hepatocytes while simultaneously impairing muscle glucose uptake, creating a pathological metabolic state that propagates systemic insulin resistance (157). Advanced imaging studies demonstrate that adipocyte-derived ceramides specifically accumulate in hepatic and muscle tissue, directly inhibiting insulin receptor substrate phosphorylation and disrupting mitochondrial function (395, 396). Key molecular executioners, particularly caspase-1 and MLKL, orchestrate distinct death programs through specific biochemical mechanisms (397). These detailed molecular insights suggest new therapeutic approaches.

Adipocyte elimination triggers extensive signaling network perturbations. Specific molecular signals from dying cells guide macrophage recruitment and inflammatory focus development. Notably, adipocyte size expansion beyond critical thresholds activates death pathways even in lean tissue by compromising phosphatidylserine-dependent clearance mechanisms. Recruited macrophages secrete elevated levels of TNF-α and IL-6 through persistent NF-κB activation (398, 399). The combination of altered adipokine profiles and disrupted adipogenic transcription compromises metabolic homeostasis (141). Continuous cycles of cell death and immune cell accumulation create an inflammatory environment that impairs insulin signaling (86). This adipose dysfunction increases lipid flux to peripheral organs, particularly through portal circulation to the liver, accelerating broader metabolic disease progression (400).

## 6 Molecular mechanisms of age-related AT dysfunction: from cellular senescence to systemic impact

Advancing age fundamentally alters AT biology, initiating molecular and cellular cascades that drive metabolic perturbations and age-associated pathologies. Primary mechanistic drivers encompass adipogenic dysregulation, cellular senescence programs, and aberrant adipokine signaling networks (401, 402). The senescence-associated secretory phenotype (SASP) emerges as a central regulator, propagating chronic inflammatory states and metabolic dysfunction (403). Age progression correlates with significant adipose depot redistribution patterns, coupled to accumulation of senescent cell populations and progressive mitochondrial deficits (404). These alterations manifest across diverse adipose-resident cell populations - mature adipocytes, immune cell subsets, and progenitor compartments (405) - culminating in systemic inflammatory activation, insulin resistance development, and accelerated aging phenotypes (406). Current therapeutic strategies targeting these age-related perturbations include senolytic compounds, nutritional interventions, physical activity protocols, and heterochronic parabiosis approaches (407).

At the molecular level, Sirtuins (silent mating type information regulation 2 homolog) emerge as crucial regulators in this complex landscape. These NAD^+^-dependent deacetylases coordinate AT function and metabolism during aging and obesity (408). Through precise regulation of lipid metabolism, inflammation, and fibrosis in AT, sirtuins fundamentally shape energy homeostasis and metabolic health (409). The aging process modulates AT function through sirtuin-dependent pathways, driving changes in fat distribution, adipogenesis, and inflammatory responses (401). SIRT1 activation enhances fatty acid oxidation and lipid mobilization, potentially protecting against obesity-linked metabolic disorders (410). Notably, obesity disrupts both adipose NAD^+^homeostasis and sirtuin enzymatic function, leading to mitochondrial deficits and metabolic complications (411).

The aging process induces dramatic changes in AT distribution and function, with significant systemic consequences. A hallmark of these changes is the reduction in subcutaneous fat accompanied by an increase in visceral fat and ectopic lipid deposition (412). The cellular composition of aging AT undergoes substantial alterations, characterized by diminished preadipocyte function and increased presence of senescent cells (401). These changes manifest in impaired adipogenesis, persistent inflammation, and dysregulated adipokine production, all contributing to insulin resistance and metabolic disorders (407). The decline in BAT activity with age further compromises metabolic homeostasis (406), although some fat redistribution patterns may serve protective functions in extreme old age (413).

Aging leads to notable alterations in the regulation of adipogenesis at the transcriptional level. Studies indicate a decline in the expression of essential adipogenic regulators such as C/EBPα and PPARγ, alongside an elevation in inhibitory factors, including C/EBPβ-LIP and CHOP (414, 415). These changes occur alongside alterations in miRNA regulation, particularly miR-143, which affects the ERK5-PPARγ axis crucial for adipocyte differentiation (416). Accumulated oxidative stress in aging AT impairs preadipocyte differentiation through cell cycle regulatory disruption (417), while enhanced SASP factor and proinflammatory cytokine production sustains chronic inflammation and insulin resistance (401).

Cellular senescence programming, characterized by permanent cell cycle arrest and SASP development, represents a fundamental mechanism driving age-related adipose dysfunction. SASP encompasses secretion of diverse factors - inflammatory mediators, growth factors, and matrix components (418) - regulated through NF-κB, C/EBPβ, and Janus kinase/Signal transducer and activator of transcription (JAK/STAT) signaling networks (419, 420). While SASP activation supports tumor suppression and tissue repair processes, it simultaneously promotes chronic inflammatory states and age-related functional decline (421). JAK pathway targeting presents therapeutic potential for addressing SASP-mediated inflammation and frailty in aging populations (422), driving research into SASP-modulating therapeutic approaches including senolytic and senomorphic compounds (423).

**Figure 1:**
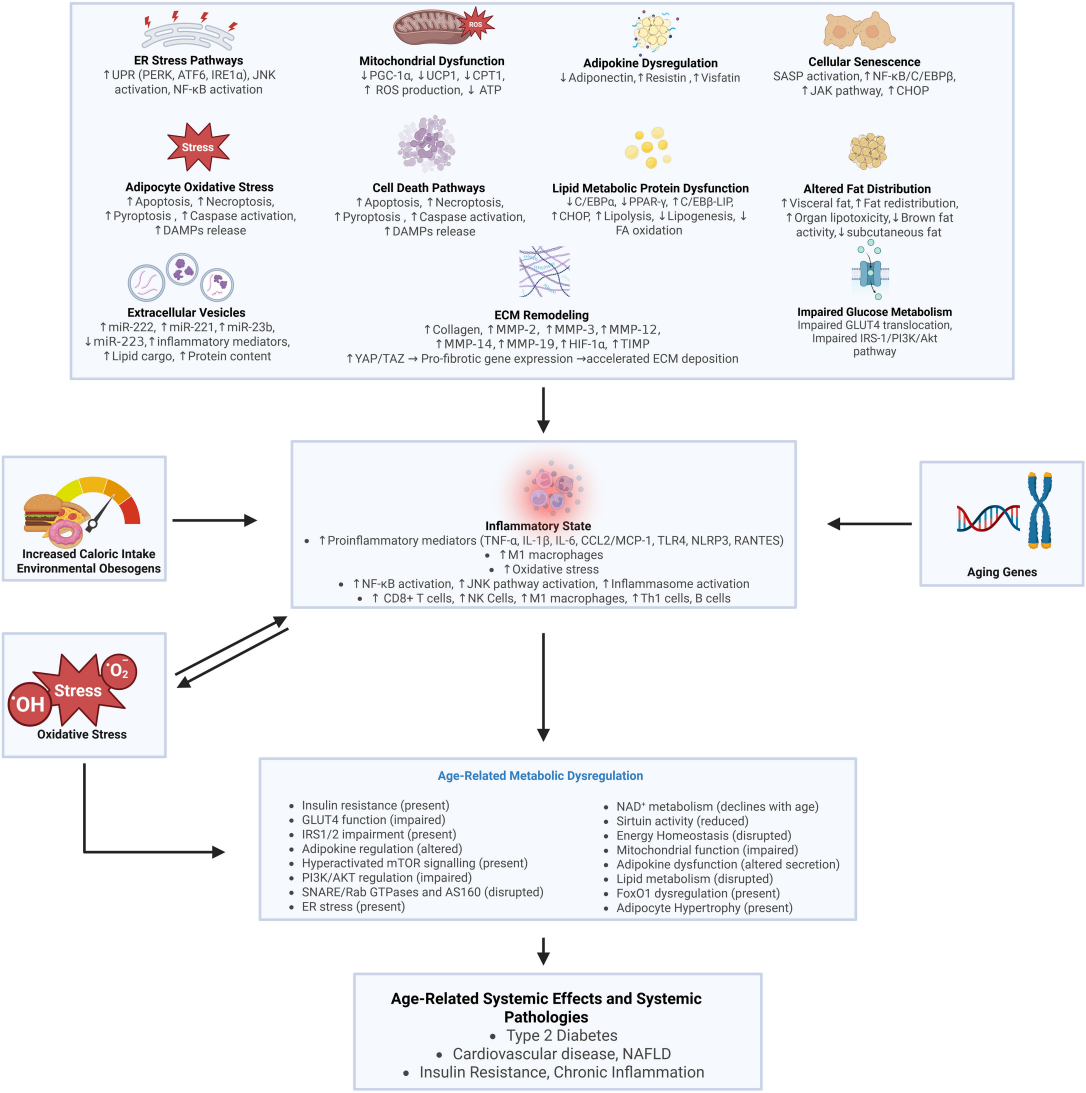
Age-related AT dysfunction arises through dysregulation of diverse molecular pathways. The schematic illustrates cellular stress responses including protein misfolding within the ER, compromised mitochondrial oxidative phosphorylation, and accelerated cellular senescence programs. Extensive ECM remodeling alters tissue architecture while impaired progenitor populations severely limit regenerative capacity. Complex metabolic perturbations emerge through increased PPAR-γ signaling cascades coupled with defective lipid oxidation pathways. Pathological fat redistribution occurs through visceral and ectopic lipid accumulation alongside progressive subcutaneous depot depletion. Adipocyte hypertrophy predominates over healthy hyperplastic expansion due to compromised progenitor function. Systemic metabolic dysregulation manifests through altered insulin sensitivity, disrupted NAD+ metabolism, and impaired mitochondrial function. The resultant disruption of glucose regulation and energy homeostasis promotes cardiometabolic disorders including T2DM and NAFLD through mechanisms spanning cellular to organismal scales. These molecular and physiological changes create integrated pathways of dysfunction affecting multiple metabolic systems.

The systemic impact of age-related AT dysfunction extends beyond local effects, contributing to widespread metabolic dysfunction and chronic low-grade inflammation (401). These changes encompass fat deposit redistribution, reduced adipogenesis, senescent cell accumulation, and altered immune cell composition (424). The dysregulated secretion of adipokines leads to insulin resistance and increased inflammation (406), while the intricate interplay between AT and the immune system becomes crucial in age-related metabolic decline, mirroring patterns observed in obesity (425). The disruption of inter-organ communication due to AT dysfunction accelerates the aging process and increases metabolic disease risk (426). Notably, both obesity and aging share key features in AT, including elevated visceral-to-subcutaneous fat ratios and pro-inflammatory immune cell phenotypes (**Figure 1**) (427).

## 7 Therapeutic advances in metabolic AT dysfunction

AT thermogenesis presents a compelling therapeutic avenue for obesity and metabolic disorders. Brown and beige adipocytes drive energy expenditure through UCP1-mediated non-shivering thermogenesis, with activation achievable via cold exposure, β3-adrenergic receptor agonists, or exercise-induced pathways (428, 429). While BAT activation demonstrates significant metabolic benefits in experimental models, sustained clinical weight loss remains challenging, and cardiovascular complications continue to limit therapeutic applications (428). Current therapeutic development encompasses multiple parallel approaches: novel pharmacological agents targeting thermogenic pathways, cell-based interventions to enhance brown adipose function, and genetic modifications designed to amplify thermogenic capacity in existing AT (430).

Adipokine signaling modulation represents another key therapeutic direction in metabolic disease treatment. Leptin sensitization strategies combat cellular resistance mechanisms, while newly developed adiponectin mimetics boost insulin sensitivity and reduce inflammatory cascades throughout metabolic tissues. The therapeutic targeting of adipokines has yielded particularly promising results for obesity-related metabolic conditions (431). These approaches span multiple molecular interventions: engineered adiponectin mimetics, synthetic leptin sensitizers, and targeted anti-inflammatory compounds, each aiming to recalibrate adipokine signaling networks and downstream metabolic parameters (432, 433). Modifying the adipose inflammasome architecture and tissue-specific inflammatory pathways shows substantial potential for attenuating chronic inflammation and restoring metabolic equilibrium across multiple tissue types.

Recent genetic and RNA-based therapeutic approaches have advanced substantially in treating metabolic dysfunction. CRISPR-Cas9 applications include generating precise lipid gene knockouts and enhancing human adipocyte browning capacity, demonstrating significant metabolic improvements in preclinical models (434). Targeted CRISPR interference against FABP4 expression in WAT reduces obesity progression and inflammatory markers in murine models (435). RNA therapeutics, including modified antisense oligonucleotides and siRNA constructs, show particular efficacy in modulating genes central to lipoprotein metabolism (436, 437). Advanced lipid nanoparticle delivery systems extend therapeutic possibilities beyond traditional hepatic targets, enabling tissue-specific intervention (438).

Diacylglycerol acyltransferase (DGAT) enzymes regulate triglyceride synthesis and lipid metabolism through multiple cellular pathways. DGAT inhibition shows therapeutic potential by limiting triglyceride synthesis and storage in metabolic tissues (439). PPARα and AMPK activation enhances fatty acid oxidation rates, reducing ectopic lipid accumulation across tissues. PPARα forms functional heterodimers with RXRβ to activate numerous genes involved in fatty acid oxidation cascades. Polyunsaturated fatty acids serve as natural PPAR activators through direct binding interactions (440). PPARβ/δ activation prevents high-fat diet-induced AMPK suppression and amplifies the PGC-1α-Lipin 1-PPARα signaling axis, substantially boosting fatty acid oxidation capacity (441). Current clinical applications include Fibrates for PPARα activation and TZDs as PPARγ agonists in dyslipidemia and diabetes treatment protocols.

Multipotent stem cells derived from AT have proven valuable in regenerative therapy. These ADSCs differentiate along distinct lineages - producing adipocytes, osteocytes, chondrocytes, and neurons under controlled conditions (442). The advantageous accessibility of ADSCs through minimally invasive lipoaspiration protocols distinguishes them from conventional mesenchymal stem cell sources (443). Their therapeutic mechanism encompasses both immunomodulatory functions and targeted trophic factor secretion, which synergistically enhance tissue repair processes (444). While clinical studies have validated ADSC efficacy across multiple pathological conditions (445), fundamental questions persist regarding lineage-specific differentiation mechanisms and delivery optimization. Elucidating the molecular determinants of cell fate decisions and characterizing the temporal dynamics of ADSC-mediated tissue regeneration remains crucial for therapeutic advancement. Contemporary investigations concentrate on decoding the complex interplay between signal transduction networks, chromatin modifications, and microenvironmental cues that regulate ADSC functionality across diverse therapeutic applications.

Nutritional interventions targeting Polyunsaturated fatty acids (PUFAs) and gut microbiota demonstrate substantial metabolic benefits through multiple mechanisms. Omega-3 PUFAs reduce AT inflammation, enhance cellular energy metabolism, and upregulate BAT thermogenic markers through direct and indirect pathways (446). These compounds modify gut microbiota composition through specific prebiotic effects on bacterial populations (447). Probiotic and prebiotic interventions favorably alter host lipid metabolism and tissue fatty acid profiles across multiple organ systems (448). Omega-3 PUFA supplementation improves circulating lipid profiles, glycemic control parameters, and hepatic fat content in various metabolic disorders. Individual genetic background and obesity status significantly influence intervention efficacy, necessitating personalized therapeutic approaches.

Molecular analyses of FGF21 and GLP-1 pathways underscore their central roles in metabolic control. Laboratory analyses of FGF21 demonstrate multifaceted effects on hepatic glucose output, AT lipid handling, and whole-body energy expenditure (449). Administration of engineered FGF21 variants yields metabolic improvements spanning glycemic control, lipid homeostasis, and body weight regulation in species ranging from mice to nonhuman primates (450). Recent clinical trials combining GLP-1 pathway activators with modified FGF21 molecules and thyroid receptor β-selective compounds show enhanced efficacy against fatty liver disease, suggesting pathway convergence in metabolic regulation (451).

The accumulation of senescent cells emerges as a critical factor in age-related metabolic decline. These cells, characterized by permanent cell cycle arrest, establish inflammatory microenvironments within adipose and other metabolic tissues. Elimination of senescent cell populations through targeted molecular approaches improves glucose homeostasis and insulin action in experimental obesity models (452). Evidence from aged mouse studies demonstrates that senescent cell removal enhances both metabolic parameters and physical function (453). Senolytic therapy shows particular promise for treating obesity-related metabolic dysfunction and numerous T2DM complications through senescent cell clearance (454, 455).

AT mitochondrial dysfunction correlates strongly with obesity progression and T2DM development, significantly affecting adipocyte differentiation capacity, cellular lipid metabolism, and insulin signaling responses. Mitochondrial function enhancement through targeted antioxidant compounds and exercise protocols improves multiple metabolic parameters (456). Complex autophagy and mitophagy pathways maintain essential adipocyte function and cellular identity. Novel antifibrotic agents target obesity-associated AT fibrosis, which contributes substantially to metabolic dysfunction through altered tissue architecture (457).

**Figure 2:**
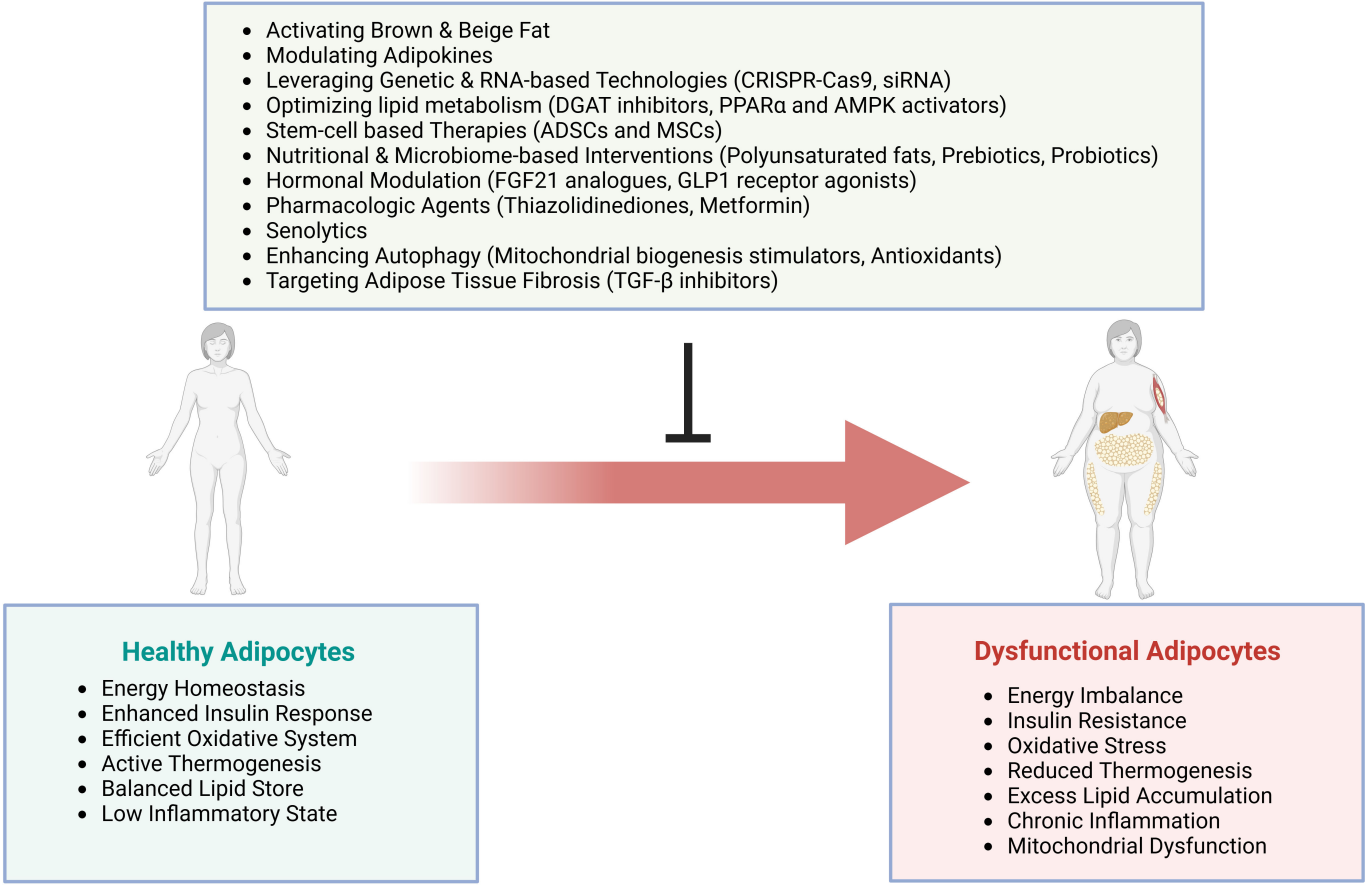
Therapeutic strategies for AT dysfunction. Key therapeutic approaches for addressing dysfunctional AT.

These therapeutic approaches target multiple fundamental aspects of adipose biology, including cellular energetics, lipid metabolism pathways, inflammatory cascades, and essential cellular maintenance processes. Combined, they represent significant advances in treatment options for metabolic diseases and systemic metabolic regulation across multiple tissue types and pathways (**Figure 2**). Current clinical investigations explore multimodal treatment combinations targeting severe metabolic disorders through integrated pathway modulation. Ongoing research continues to reveal additional molecular targets and regulatory pathways crucial for metabolic disease treatment strategies.

## 8 Conclusion

AT dysfunction disrupts metabolic homeostasis through complex interactions of cellular stress, inflammatory processes, and regulatory mechanisms. Our analysis reveals critical molecular pathways, including NF-κB and JNK signaling, that fundamentally compromise metabolic function.

Inflammatory pathways and oxidative stress critically impact metabolic health. Specific molecular markers like TNF-α, IL-6, and increased ROS demonstrate how AT depots generate unique cellular responses that influence systemic metabolism.

Molecular alterations in receptor functionality, particularly IR signaling and GLUT4 translocation, generate metabolic disruptions through complex inter-organ communication. Adipokine interactions, including dysregulated leptin and adiponectin profiles, reveal complex signaling networks between AT and metabolic systems.

Emerging therapeutic strategies target specific molecular mechanisms, including PPAR-γ pathway modulation, BAT activation through UCP1 targeting, AMPK pathway interventions, senolytic approaches to eliminate dysfunctional adipose cells, and precise genomic and RNA-based interventions.

Critical knowledge gaps persist in our understanding of AT dysfunction, particularly regarding depot-specific molecular heterogeneity, temporal dynamics of dysfunction progression, and sex-specific differences in adipose pathophysiology. The field requires comprehensive single-cell resolution mapping of AT microenvironments, longitudinal studies tracking dysfunction development from early metabolic stress to established disease, and systems-level integration of multi-omics data to identify causal relationships in metabolic regulation.

Key research priorities include: (1) elucidating the molecular determinants of adipose depot specialization and their therapeutic potential; (2) characterizing the temporal sequence of cellular events during dysfunction progression to identify critical intervention windows; (3) investigating sex hormone influences on adipose immune cell trafficking and inflammatory resolution; (4) developing novel imaging technologies for non-invasive assessment of AT health; and (5) designing targeted delivery systems for adipose-specific therapeutic agents that avoid systemic effects.

Future investigations should prioritize translational approaches bridging mechanistic discoveries to clinical applications. Personalized medicine strategies incorporating AT biomarkers, genetic risk profiles, and metabolic phenotyping may enable early intervention before irreversible dysfunction occurs. Integration of artificial intelligence with multi-omics analyses could reveal previously unrecognized regulatory networks and therapeutic targets. Ultimately, addressing these knowledge gaps through coordinated research efforts will advance our ability to combat the global epidemic of metabolic disease.
